# Botensilimab plus balstilimab in relapsed/refractory microsatellite stable metastatic colorectal cancer: a phase 1 trial

**DOI:** 10.1038/s41591-024-03083-7

**Published:** 2024-06-13

**Authors:** Andrea J. Bullock, Benjamin L. Schlechter, Marwan G. Fakih, Apostolia M. Tsimberidou, Joseph E. Grossman, Michael S. Gordon, Breelyn A. Wilky, Agustin Pimentel, Daruka Mahadevan, Ani S. Balmanoukian, Rachel E. Sanborn, Gary K. Schwartz, Ghassan K. Abou-Alfa, Neil H. Segal, Bruno Bockorny, Justin C. Moser, Sunil Sharma, Jaymin M. Patel, Wei Wu, Dhan Chand, Katherine Rosenthal, Gabriel Mednick, Chloe Delepine, Tyler J. Curiel, Justin Stebbing, Heinz-Josef Lenz, Steven J. O’Day, Anthony B. El-Khoueiry

**Affiliations:** 1 Beth Israel Deaconess Medical Center, Boston, MA USA; 2 Dana-Farber Cancer Institute, Boston, MA USA; 3 City of Hope Comprehensive Cancer Center, Duarte, CA USA; 4 The University of Texas MD Anderson Cancer Center, Houston, TX USA; 5 Agenus, Inc., Lexington, MA USA; 6 HonorHealth Research Institute, Scottsdale, AZ USA; 7 University of Colorado Cancer Center, Aurora, CO USA; 8 Sylvester Comprehensive Cancer Center, University of Miami, Miami, FL USA; 9 The University of Texas Health Science Center at San Antonio, San Antonio, TX USA; 10 The Angeles Clinic and Research Institute, Los Angeles, CA USA; 11 Earle A. Chiles Research Institute, Providence Cancer Institute, Portland, OR USA; 12 Case Comprehensive Cancer Center, Case Western Reserve University, Cleveland, OH USA; 13 Memorial Sloan Kettering Cancer Center, New York, NY USA; 14 Weill Medical College at Cornell University, New York, NY USA; 15 Trinity College Dublin, Dublin, Ireland; 16 Dartmouth Cancer Center, Lebanon, NH USA; 17 Anglia Ruskin University, Cambridge, UK; 18 University of Southern California Norris Comprehensive Cancer Center, Los Angeles, CA USA; 19 Providence Saint John’s Cancer Institute, Santa Monica, CA USA

**Keywords:** Colorectal cancer, Cancer immunotherapy

## Abstract

Microsatellite stable metastatic colorectal cancer (MSS mCRC; mismatch repair proficient) has previously responded poorly to immune checkpoint blockade. Botensilimab (BOT) is an Fc-enhanced multifunctional anti-cytotoxic T-lymphocyte-associated protein 4 (CTLA-4) antibody designed to expand therapy to cold/poorly immunogenic solid tumors, such as MSS mCRC. BOT with or without balstilimab (BAL; anti-PD-1 antibody) is being evaluated in an ongoing expanded phase 1 study. The primary endpoint is safety and tolerability, which was evaluated separately in the dose-escalation portion of the study and in patients with MSS mCRC (using combined dose-escalation/dose-expansion data). Secondary endpoints include investigator-assessed RECIST version 1.1–confirmed objective response rate (ORR), disease control rate (DCR), duration of response (DOR) and progression-free survival (PFS). Here we present outcomes in 148 heavily pre-treated patients with MSS mCRC (six from the dose-escalation cohort; 142 from the dose-expansion cohort) treated with BOT and BAL, 101 of whom were considered response evaluable with at least 6 months of follow-up. Treatment-related adverse events (TRAEs) occurred in 89% of patients with MSS mCRC (131/148), most commonly fatigue (35%, 52/148), diarrhea (32%, 47/148) and pyrexia (24%, 36/148), with no grade 5 TRAEs reported and a 12% discontinuation rate due to a TRAE (18/148; data fully mature). In the response-evaluable population (*n* = 101), ORR was 17% (17/101; 95% confidence interval (CI), 10–26%), and DCR was 61% (62/101; 95% CI, 51–71%). Median DOR was not reached (NR; 95% CI, 5.7 months–NR), and median PFS was 3.5 months (95% CI, 2.7–4.1 months), at a median follow-up of 10.3 months (range, 0.5–42.6 months; data continuing to mature). The combination of BOT plus BAL demonstrated a manageable safety profile with no new immune-mediated safety signals and encouraging clinical activity with durable responses. ClinicalTrials.gov identifier: NCT03860272.

## Abstract (web summary)

In an ongoing phase 1 trial, the combination of two new immunotherapies targeting CTLA-4 and PD-1 was overall well tolerated and elicited encouraging clinical responses in patients with relapsed/refractory microsatellite stable colorectal cancer, a tumor type typically unresponsive to immune checkpoint blockade.

## Main

Colorectal cancer (CRC) is the second leading cause of cancer death in the United States, comprising an estimated 8.3% of cancer-related deaths annually^1^. Although overall mortality from CRC has declined, survival remains poor for advanced disease, and the burden is shifting to a younger population^2^. Alarmingly, from 1995 to 2019, the number of patients under the age of 55 who were diagnosed with CRC in the United States nearly doubled^3^.

Chemotherapy remains the cornerstone of treatment, and although survival has increased with the addition of monoclonal antibodies targeting vascular endothelial growth factor (VEGF) and epidermal growth factor receptors (EGFRs), improvements have been moderate^4–8^. Immunotherapy using checkpoint inhibitors targeting programmed cell death (ligand) 1 (PD-(L)1) and cytotoxic T-lymphocyte-associated protein 4 (CTLA-4) have become established pillars of systemic therapy across oncology, often achieving durable remissions. However, response rates vary across tumor types, with particularly disappointing outcomes in patients with metastatic colorectal cancer (mCRC) that is not microsatellite instability-high (MSI-H) or mismatch repair deficient (dMMR) and is commonly referred to as microsatellite stable (MSS) or mismatch repair proficient^9–12^. Since their initial approval in metastatic melanoma^13^, immune checkpoint blockade (ICB) regimens have demonstrated efficacy but only gained regulatory approval in select gastrointestinal cancers, such as gastroesophageal cancer and, more recently, cholangiocarcinoma^14,15^.

The only patients with mCRC for whom ICB has been shown to be efficacious and is approved are the approximately 4% with MSI-H or dMMR tumors, in whom response rates are around 40% and higher, likely due to increased neoantigen load^16–18^. Conversely, response rates to PD-(L)1 inhibition in MSS mCRC are near 0%, and immunotherapy-only combinations including PD-(L)1/CTLA-4 have also demonstrated limited efficacy^19^. The combination of durvalumab (anti-PD-1 antibody) and tremelimumab (anti-CTLA-4 antibody) was evaluated in a randomized phase 2 study in patients with mCRC and resulted in one objective response out of 119 patients, a median progression-free survival (PFS) of 1.8 months and a median overall survival (OS) of 6.6 months. Similarly, nivolumab (anti-PD-1 antibody) and ipilimumab (anti-CTLA-4 antibody) were evaluated in two separate trials with disappointing efficacy: the TAPUR phase 2 basket study examined individuals in whom all lines of therapy had been exhausted and reported results from patients with mCRC with high tumor mutational burden (TMB; defined as ≥9 mutations per megabase (Mut/Mb)) treated with nivolumab and ipilimumab^20^. There was only one response out of 10 patients treated, a median PFS of 3.1 months and a median OS of 9.9 months. Similarly, in the CheckMate-142 study, only one of 23 patients with MSS mCRC treated with ipilimumab and nivolumab responded^16^.

MSS mCRC can be classified as poorly immunogenic or ‘cold’, with a tumor microenvironment (TME) commonly infiltrated by immunosuppressive regulatory T cells (T_regs_) and myeloid cells and characteristically absent of CD8^+^ effector T cells (T_effectors_)^17^. A multitude of tumor-intrinsic and tumor-extrinsic factors, such as low TMB and dysfunctional adaptive immunity, for example, may contribute to refractoriness to ICB and resistance to other primary and adaptive immunotherapies in MSS mCRC^17,18,21,22^.

Fragment crystallizable gamma receptor (FcγR) interactions have been shown to play a central role in antibody-directed immune effector cell activities. For anti-CTLA-4 antibodies, the interaction with FcγR on antigen-presenting cells (APCs) and natural killer (NK) cells enhances antigen-specific T cell responses and tumoricidal activity^23^. Furthermore, human FcγRIIIA polymorphisms, along with neoantigen burden, have been shown to impact responses to ipilimumab in patients with advanced melanoma^24^.

Botensilimab (AGEN1181; BOT) is a novel multifunctional Fc-enhanced anti-CTLA-4 antibody intentionally designed to overcome the limitations of conventional ICB and address the unmet need in tumors classified as ‘cold’ or refractory to prior ICB. In addition to complete blockade of CTLA-4 ligand interactions, the Fc-enhanced IgG1 region of BOT binds with increased binding affinity to activating FcγRs on APCs and NK cells, including both FcγRIIIA variants, low-affinity F158 and high-affinity V158, compared to conventional IgG1 anti-CTLA-4 antibodies (for example, ipilimumab). Therefore, in addition to blocking CTLA-4, BOT promotes optimized T cell priming, activation and memory formation by strengthening APC and T cell co-engagement^23^. As an Fc-enhanced anti-CTLA-4 antibody, BOT also promotes intratumoral T_reg_ depletion via antibody-dependent cellular cytotoxicity and phagocytosis mechanisms. These qualities differentiate BOT from approved CTLA-4 antibodies with the potential for BOT to extend benefit to ‘cold’ or ICB-refractory tumors by activating both the innate and adaptive immune system^25,26^. Balstilimab (AGEN2034; BAL) is a fully human monoclonal antibody that binds with high affinity to PD-1, preventing the interaction between the receptor and its ligands PD-L1 and/or PD-L2 with a similar efficacy and safety profile to currently approved PD-1 inhibitors^27^.

A phase 1, open-label study of BOT with or without BAL was initiated in multiple relapsed/refractory (R/R) solid tumors (NCT03860272) to assess safety and tolerability as well as to test if the specific design of BOT translates into clinical benefit. The study began with the dose escalation of BOT monotherapy and then the BOT and BAL combination, followed by disease-specific expansion cohorts, including one that enrolled patients with MSS mCRC for treatment with BOT monotherapy or combination therapy. Here we report safety and preliminary efficacy outcomes for the heavily pre-treated MSS mCRC patient population treated in this phase 1 study.

## Results

### Dose escalation

**Fig. 1:**
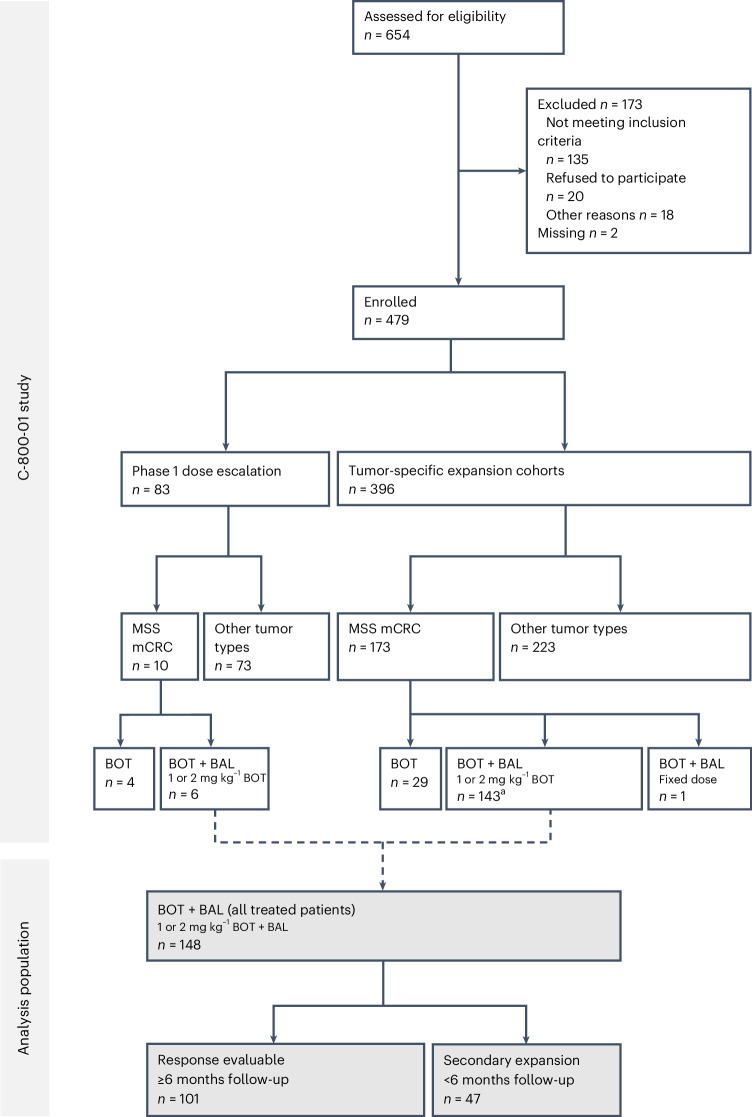
Participant flow diagram of the C-800-01 study and all treated patients with MSS mCRC (*n* = 148; analysis population). ^a^ One patient was enrolled but was never treated.

**Extended Data Fig. 1:**
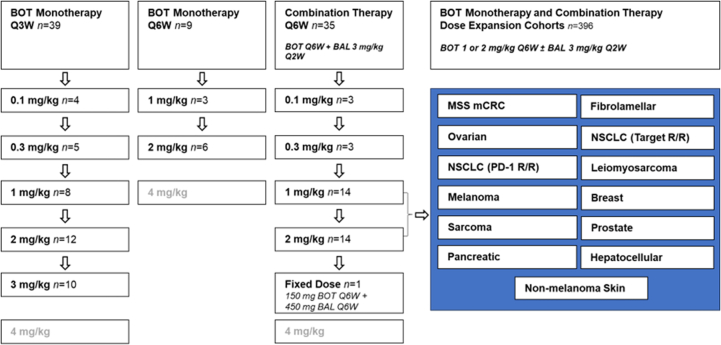
Study schema for enrolled patients in the dose-escalation or dose expansion (*N*=479). NSCLC; non-small cell lung cancer; PD-1, programmed cell death protein 1; Q3W, every 3 weeks; R/R relapsed/refractory.

The dose-escalation portion of the C-800-01 study (3 + 3 design) occurred from 1 April 2019 to 31 August 2023 (data cutoff: 29 November 2023), whereby 83 patients with advanced solid tumors (including 10 patients with MSS mCRC) were enrolled and treated with BOT monotherapy every 3 weeks (Q3W) or 6 weeks (Q6W; staggered with Q3W dosing), starting at dose level 0.1 mg kg^−1^ up to 3 mg kg^−1^, administered intravenously (IV) for up to 2 years, or in combination with BAL 3 mg kg^−1^ every 2 weeks (Q2W), administered IV for up to 2 years. Patients could be treated beyond progression. A total of 48 patients were treated with BOT monotherapy, and 35 patients received the combination of BOT plus BAL (Fig. 1 and Extended Data Fig. 1). Baseline demographics and disease characteristics for all dose-escalation cohorts are summarized in Extended Data Table 1.

There were no dose-limiting toxicities (DLTs) in either BOT monotherapy or BOT plus BAL combination therapy dose-escalation cohorts, which was the primary endpoint of the study. A maximum tolerated dose was not reached, and further investigation is warranted to determine the recommended phase 2 dose (RP2D) by disease indication. Both monotherapy and combination therapy dose-escalation cohorts received a median of two BOT doses (range, 1–23 BOT monotherapy; range, 1–17 combination therapy; Supplementary Tables 1 and 2). A secondary endpoint was assessing the severity and causality of treatment-emergent adverse events (TEAEs) for all dose groups. Treatment-related adverse events (TRAEs) of any grade, defined as TEAEs related to BOT or BAL, occurred in 75% of monotherapy patients (36/48; 33% grade ≥3) and in 83% of combination therapy patients (29/35; 26% grade ≥3). Additional safety data from the dose-escalation portion of the study are summarized in Supplementary Tables 3 and 4.

Other secondary endpoints include objective response rate (ORR), disease control rate (DCR, defined as a best response of complete response (CR), partial response (PR) or stable disease (SD) for at least 6 weeks) and progression-free survival (PFS) for all dose groups per Response Evaluation Criteria in Solid Tumors version 1.1 (RECIST 1.1), based on investigator assessment, as well as pharmacokinetics and immunogenicity. Imaging was performed Q6W (±3 d). Objective responses were observed in eight of 83 dose-escalation patients (10%; 95% confidence interval (CI), 4–18%). Among monotherapy patients, a patient with PD-1 R/R endometrial carcinoma achieved a CR, and a patient with PD-1 R/R cervical cancer achieved a PR. In the combination dose-escalation cohorts, six patients achieved a PR—three with MSS mCRC, two with platinum resistant/refractory ovarian cancer and one with endometrial carcinoma. An additional 28 patients (34%) had SD, and the DCR was 43% (95% CI, 33–55%) as of the data cutoff (29 November 2023). Median follow-up was 6.3 months (range, 0.7–42.6 months), and the median duration of response (DOR) was not reached (NR; 95% CI, 4.2 months–NR). Additional efficacy analyses in all dose-escalation cohorts are provided in Extended Data Table 2. As part of a protocol amendment, patients were permitted to cross over from BOT monotherapy to the combination upon progression. Out of 13 total patients who first received BOT monotherapy and later crossed over to the combination, there were four responders (two patients with MSS mCRC, one with dedifferentiated liposarcoma and one with platinum resistant/refractory ovarian cancer).

In a separate pharmacokinetic and immunogenicity analysis, BOT exhibited dose-proportional increases in exposure metrics (maximum concentration (C_max_), minimum concentration (C_min_) and trough concentration (C_trough_)) across the dose range of 0.1 mg kg^−1^ to 3 mg kg^−1^ for monotherapy and 0.1 mg kg^−1^ to 2 mg kg^−1^ in combination with BAL. After administration of a single dose of BOT at 1 mg kg^−1^ Q6W, geometric mean C_max_ was 22.64 μg ml^−1^ for monotherapy and 19.08 μg ml^−1^ in combination with BAL. At steady state, geometric mean C_max_ was 23.86 μg ml^−1^ for monotherapy and 20.12 μg ml^−1^ in combination with BAL at the same dose level. The estimated geometric mean terminal half-life of BOT was approximately 14 d. In addition, in a small number of patients who were positive for antidrug antibodies (ADAs), the potential impact on BOT pharmacokinetics was assessed. Exposure levels were similar between patients who were ADA positive or negative.

### MSS mCRC patient characteristics

Patients were subsequently enrolled in select disease-specific expansion cohorts as the dose escalation was completed, including MSS mCRC, sarcomas, ovarian cancer, PD-1 R/R melanoma, PD-1 R/R hepatocellular carcinoma, PD-1 R/R non-small cell lung cancer and others (Fig. 1 and Extended Data Fig. 1). As of the data cutoff (29 November 2023), a total of 148 patients with MSS mCRC were treated with BOT 1 mg kg^−1^ or 2 mg kg^−1^ Q6W plus BAL 3 mg kg^−1^ Q2W (with prior ICB permitted). Of note, 10 patients with MSS mCRC were included in the dose-escalation portion of the study—four who received BOT monotherapy (0.3 mg kg^−1^ Q3W, 1 mg kg^−1^ Q3W, 1 mg kg^−1^ Q6W and 2 mg kg^−1^ Q6W) and six who received the BOT and BAL combination (two who received 1 mg kg^−1^ BOT and four who received 2 mg kg^−1^ BOT); and 142 patients were from the MSS mCRC expansion cohort (an additional patient was enrolled but never received treatment). In this population, approximately 79% of responses to BOT and BAL are observed by 6 months (excluding patients who left the study early). Based on these response kinetics, 101 with at least 6 months of follow-up out of the total 148 patients with MSS mCRC treated with 1 mg kg^−1^ or 2 mg kg^−1^ of BOT and BAL were considered response evaluable (Fig. 1).

Baseline demographics and disease characteristics are summarized in Table 1 for all treated patients (and broken down by response-evaluable patients (*n* = 101) and additional treated patients (*n* = 47) in Extended Data Table 3). Median age was 56 years (range, 25–82). Seventy-six patients (51%) were female, and 79 (53%) had an Eastern Cooperative Oncology Group (ECOG) performance status of 1. This patient population was heavily pre-treated with a median of three prior lines of therapy (range, 1–10), and 24 of 148 patients (16%) had received prior PD-(L)1 inhibitors (three of whom also received prior CTLA-4 inhibition when prior protocol versions allowed). There were 25 patients (17%) with active liver metastases (LM) and 123 patients (83%) with no active liver metastases (NLM). Patients with NLM were further broken down into patients with treated LM (20 patients (14%)), defined as having LM that were resected, ablated or treated with stereotactic body radiation therapy without recurrence and patients with no history of LM (103 patients (70%)). One hundred patients (68%) had more than one site of metastatic disease, and 42% had peritoneal disease. Of 40 patients in whom TMB data from central assessment were readily available, only one had a TMB > 10 Mut/Mb (TMB = 13 Mut/Mb; via whole-exome sequencing (WES); Personalis ImmunoID NeXT assay). Of the 45 patients in whom mutation data were available, 26 (58%) harbored *RAS* mutations, and two (4%) had *BRAF* mutations (as detected with the Personalis ImmunoID NeXT assay).

**Table 1:** Baseline demographics and disease characteristics in all treated patients with MSS mCRC (*N* = 148)

	Patients with MSS mCRC (*N* = 148)
Median age, years (range)	56 (25–82)
Female, *n* (%)	76 (51)
ECOG performance status at baseline, *n* (%)	
0	69 (47)
1	79 (53)
Median prior lines of therapy (range)	3 (1–10)
Prior PD-(L)1/CTLA-4, *n* (%)	24 (16)
Prior regorafenib, *n* (%)	30 (20)
Prior trifluridine/tipiracil, *n* (%)	24 (16)
Prior regorafenib as well as trifluridine/tipiracil, *n* (%)	43 (29)
Prior bevacizumab, *n* (%)	120 (81)
Prior bevacizumab/trifluridine/tipiracil, *n* (%)	14 (9)
Prior radiotherapy, *n* (%)	64 (43)
Multiple metastatic sites, *n* (%)	100 (68)
Peritoneal disease, *n* (%)	62 (42)
BOT dose, *n* (%)	
1 mg kg^−1^ Q6W + BAL (PD-1) Q2W	67 (45)
2 mg kg^−1^ Q6W + BAL (PD-1) Q2W	81 (55)
Liver involvement, *n* (%)	
Active LM	25 (17)
NLM	123 (83)
Treated LM	20 (14)
No history of LM	103 (70)
TMB > 10, *n/N* (%)^1^	1/40 (3)
*RAS* mutation, *n* (%)^1^	26/45 (58)
*BRAF* mutation, *n/N* (%)^1^	2/45 (4)

^1^ TMB status was unknown in 108 patients, whereas *RAS* and *BRAF* statuses were unknown in 103 patients.

### Safety

In addition to all patients in the dose-escalation portion of the study (*n* = 83; Supplementary Tables 3 and 4), the 148 patients with MSS mCRC treated with BOT at 1 mg kg^−1^ or 2 mg kg^−1^ in combination with BAL (including the six patients from the dose-escalation portion of the study and 142 patients from the dose-expansion portion) were evaluable for safety (with fully mature data). TRAEs of any grade occurred in 89% (131/148) of patients, most commonly fatigue (35%; 52/148; 1% grade 3, no grade 4), diarrhea (32%; 47/148; 5% grade 3, no grade 4) and pyrexia (24%; 36/148; 3% grade 3, no grade 4). Multiple events could occur in the same patient (Table 2 and Supplementary Table 5). Fifty-two patients (35%) encountered a serious TRAE (22% grade 3, 1% grade 4); 47 patients (32%) encountered a treatment-related study drug interruption (7% grade 3, <1% grade 4); 42 patients (28%) discontinued any study drug due to a TRAE (16% grade 3, no grade 4); and 18 patients (12%) discontinued both BOT and BAL due to a TRAE. Immune-mediated TRAEs were defined as having been treated with steroids of any dose or other immunosuppressants, related to treatment, and comprised multiple preferred terms, occurring in 49% (72/148) of patients. The immune-mediated TRAE of ‘diarrhea/colitis’ comprised preferred terms of diarrhea, colitis and enteritis and was the most common occurring in 33% of patients (49/148; 10% grade 3, <1% grade 4). Other common immune-mediated TRAEs included skin reactions (18%; 26/148; no grade 3 or grade 4) and hepatitis (12%; 18/148; 3% grade 3, no grade 4; Supplementary Table 6). As of the data cutoff, 41 patients (28%) remained on treatment, and 78 patients (53%) remained on study.

**Table 2:** Any-grade TRAEs in at least 5% of all treated patients with MSS mCRC (*n* = 148)

	Grade 1	Grade 2	Grade 3	Grade 4	All-grade
Any TRAE^1^, *n* (%)	27 (18)	56 (38)	46 (31)	2 (1)	131 (89)
Fatigue	27 (18)	23 (16)	2 (1)	0	52 (35)
Diarrhea	18 (12)	21 (14)	8 (5)	0	47 (32)
Pyrexia	17 (11)	15 (10)	4 (3)	0	36 (24)
Decreased appetite	21 (14)	12 (8)	0	0	33 (22)
Chills	29 (20)	1 (<1)	0	0	30 (20)
Pruritis	21 (14)	7 (5)	0	0	28 (19)
Nausea	20 (14)	5 (3)	2 (1)	0	27 (18)
Colitis	3 (2)	18 (12)	4 (3)	1 (<1)	26 (18)
Rash maculopapular	18 (12)	7 (5)	0	0	25 (17)
Arthralgia	16 (11)	5 (3)	0	0	21 (14)
Alanine aminotransferase increased	11 (7)	6 (4)	2 (1)	0	19 (13)
Anemia	8 (5)	8 (5)	0	0	16 (11)
Headache	14 (9)	1 (<1)	1 (<1)	0	16 (11)
Myalgia	10 (7)	6 (4)	0	0	16 (11)
Immune-mediated enterocolitis	1 (<1)	7 (5)	6 (4)	0	14 (9)
Rash	10 (7)	4 (3)	0	0	14 (9)
Vomiting	9 (6)	4 (3)	1 (<1)	0	14 (9)
Aspartate aminotransferase increased	8 (5)	3 (2)	2 (1)	0	13 (9)
Hyperhidrosis	10 (7)	1 (<1)	0	0	11 (7)
Stomatitis	5 (3)	5 (3)	0	0	10 (7)
Blood alkaline phosphatase increased	6 (4)	2 (1)	0	0	8 (5)
Dyspnea	4 (3)	4 (3)	0	0	8 (5)
Blood creatine phosphokinase increased	2 (1)	0	4 (3)	1 (<1)	7 (5)
Blood creatinine increased	4 (3)	3 (2)	0	0	7 (5)
Dizziness	6 (4)	1 (<1)	0	0	7 (5)
Dry mouth	7 (5)	0	0	0	7 (5)
Hypothyroidism	1 (<1)	6 (4)	0	0	7 (5)

1 TEAEs related to BOT or BAL were coded using the MedDRA, version 22.1, classified by system organ class and preferred term. TRAEs (TEAEs related to BOT or BAL) were defined as AEs with onset dates or the worsening of an event during the extended on-treatment period, which was defined as time from the first dose of study treatment to last dose of study treatment +90 d or the earliest date of new anti-cancer therapy –1 d, whichever occurred first.

Grade 3 TRAEs occurred in 31% of patients (46/148) and included eight cases (5%) of diarrhea; six cases of immune-mediated enterocolitis (4%); four cases each (3%) of colitis and pyrexia; three cases of acute kidney injury (2%); and two cases each (1%) of adrenal insufficiency, fatigue, immune-mediated hepatitis and nausea. The grade 3 TRAEs of immune-mediated hypophysitis, immune-mediated myocarditis and pneumonitis were rare, occurring in less than 1% of patients. Grade 4 TRAEs occurred in 1% of patients (2/148) and included one case each (<1%) of colitis and blood creatine phosphokinase increased. There were no treatment-related deaths (grade 5 events).

Based on data supporting the use of tumor necrosis factor (TNF) inhibitors in the steroid-sensitive setting and clinical practice, infliximab was used to manage the immune-mediated TRAE of diarrhea/colitis in this study even in the non-refractory low-grade setting^28,29^. Patients who received infliximab were able to continue on study drug if toxicity resolved and was not higher than grade 2. If a patient had a grade 3 event related to BOT but not to BAL, they were allowed to continue BAL. Of 36 total patients who received immunosuppressants for an immune-mediated TRAE of diarrhea/colitis, 35 received infliximab (97%), and 31 received concurrent steroids (86%). Nineteen of these 36 patients (30 events; four grade 1 events, 13 grade 2 events, two grade 3 events) either temporarily stopped BOT or remained on treatment. Thirty-one of those 36 patients had complete resolution of diarrhea/colitis, and five patients had unresolved diarrhea/colitis (two with sequelae), suggesting that early treatment with infliximab can effectively manage immune-mediated treatment-related diarrhea/colitis^29^. Other TRAEs were managed according to guidelines at the investigator’s discretion^30^.

### Efficacy

**Fig. 2:**
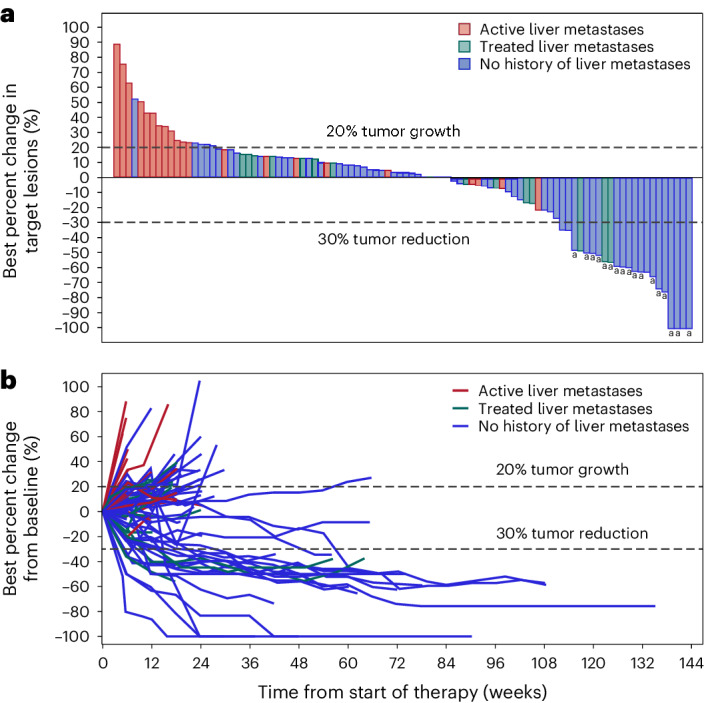
Clinical efficacy by liver involvement in response-evaluable patients with MSS mCRC (*n* = 101). Liver involvement was characterized as patients with active LM, treated LM that were resected or ablated without recurrence or no history of LM. **a**, Best overall response. **b**, Response over time. ^a^ Indicates a RECIST 1.1–confirmed CR or PR. The first blue bar on the right represents the CR.

**Fig. 3:**
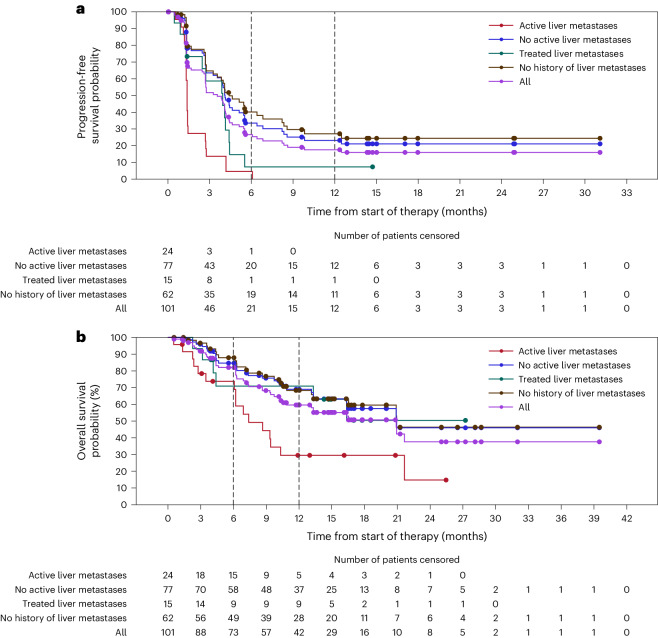
Clinical outcomes by liver involvement in response-evaluable patients with MSS mCRC (*n* = 101). Liver involvement was characterized as patients with active LM, treated LM that were resected or ablated without recurrence or no history of LM. No active LM included patients with treated LM together with patients with no history of LM. **a**, PFS. **b**, OS.

Of the 148 treated patients with MSS mCRC, 101 with at least 6 months of follow-up were evaluated in efficacy analyses (including four patients from the dose-escalation portion of the study and 97 from the dose-expansion portion; data on the additional 47 treated patients are continuing to mature). Objective responses were observed in 17 of 101 patients (17%; 95% CI, 10–26%), including one CR (Fig. 2a). An additional 45 patients (45%) had SD, and the DCR was 61% (95% CI, 51–71%; Fig. 2b). The clinical benefit rate (CBR, defined as best response of CR, PR or SD for at least 24 weeks) was 28% (95% CI, 19–38%). The median DOR was NR (95% CI, 5.7 months–NR), and the median PFS was 3.5 months (95% CI, 2.7–4.1 months) (Fig. 3a). A final exploratory endpoint assessed median OS and/or rate of OS. The median OS was 20.9 months (95% CI, 10.6 months–NR) (Fig. 3b), and the 12-month OS rate was 60% (95% CI, 49–69%), with a median follow-up of 10.3 months (range, 0.5–42.6 months). Eleven of the 17 responding patients had ongoing responses at the time of the data cutoff. An additional PR was confirmed after the data cutoff (18/101; ORR 18%). Five patients had at least 30% reduction in target lesions, as noted in Fig. 2a, although these responses did not qualify as RECIST-confirmed responses. One patient later had progressive disease (PD); one received surgery at 20 weeks and was unevaluable; one initially had a response until 60 weeks that was changed to a best response of SD after a small peri-splenic nodule at week 66 was observed that was retrospectively identified as being present since week 18 (with all other lesions remaining in a response); one had a RECIST response at 6 weeks and had a surgical resection of a target lesion that was considered clinical progression based on increase from nadir at 12 weeks (after receiving steroids for an immune-mediated TRAE) and was not confirmed, the remaining target lesions then progressed; and another patient had a PR in target lesions but experienced recurrence of treated LM at week 18. In the additional 47 treated patients with fewer than 6 months of follow-up, several objective responses have been observed thus far, although the median follow-up for these patients is only 3.3 months (range, 1.9–4.9 months). Updates will be provided as the data reach maturity.

### Analysis by liver involvement

An exploratory analysis by liver involvement was conducted early in the study, and more favorable outcomes were observed in patients with NLM (Figs. 2a,b and 3a,b). The protocol was subsequently amended to focus on patients with NLM to reduce heterogeneity and complexity in the phase 1 patient population.

In patients with NLM (*n* = 77), the ORR was 22% (17/77; 95% CI, 13–33%); the DCR was 73% (95% CI, 61–82%); the CBR was 35% (95% CI, 25–47%); the median DOR was NR (95% CI, 5.7 months–NR); and the median PFS was 4.1 months (95% CI, 3.3–5.5 months) (Fig. 3a). The median OS was 20.9 months (95% CI, 16.4 months–NR), and the 12-month OS rate was 69% (95% CI, 56–79%), with a median follow-up of 13.0 months (range, 0.6–42.6 months) (Fig. 3b). As mentioned previously, an additional response was confirmed after the data cutoff in a patient with NLM (18/77; ORR 23%). Among the 77 patients with NLM, 16 had previously treated LM, and two of these 16 patients had a PR.

In patients with active LM (*n* = 24), the ORR was 0% (0/24; 95% CI, 0–14%); the DCR was 25% (95% CI, 10–47%); the CBR was 4% (95% CI, 0–21%); and the median PFS was 1.4 months (95% CI, 1.3–1.4 months) (Fig. 3a). The median OS was 7.4 months (95% CI, 6.1–10.3 months), and the 12-month OS rate was 30% (95% CI, 12–49%), with a median follow-up of 6.2 months (range, 0.5–21.7 months) (Fig. 3b).

### Biomarker and gene expression analysis

Exploratory endpoints in this study included investigating biomarkers that may predict pharmacologic activity or response to BOT with or without BAL and exploring immune biomarkers in peripheral blood and tumor tissue. Thus, pre-treatment tumor tissue was analyzed to characterize potential genomic and biomarker differences among responders, patients with SD (of at least 12 weeks) and those with PD (per RECIST 1.1). Local and central genomics testing with the Personalis ImmunoID NeXT assay was available for nine of the 17 responders. Only one had a TMB ≥ 10 Mut/Mb at 13 Mut/Mb; four had *RAS* mutations (*KRAS*^*G12D*^, *KRAS*^*G13D*^, *NRAS*^*Q61R*^ and *KRAS*^*Q61L*^); and one had a *BRAF*^*G466V*^ mutation.

**Fig. 4:**
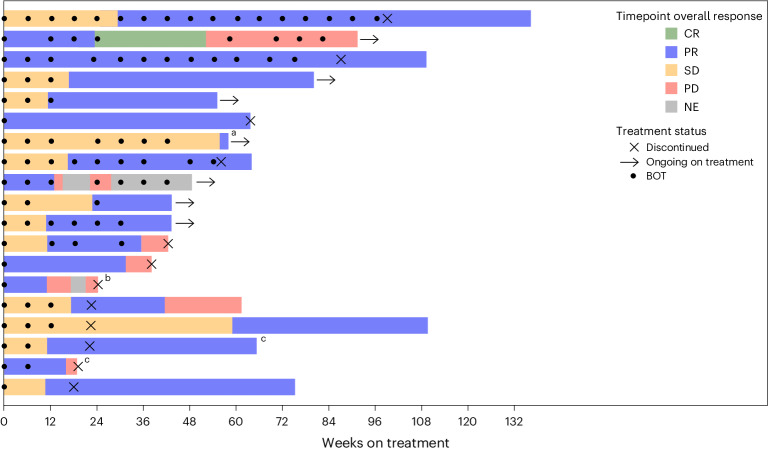
Responder characteristics (*n* = 19). NLM was defined as patients with no history of LM (*n* = 17) or patients whose LM were resected or ablated without recurrence (treated; *n* = 2). Arrows indicate patients who, at the time of the data cutoff, were ongoing on treatment. ^a^ One patient later progressed. ^b^ One patient with a RECIST 1.1 response at 6 weeks had surgical resection of a target lesion that was considered clinical progression based on increase from nadir at 12 weeks after receiving steroids for an immune-mediated TRAE and was not confirmed; remaining target lesions then progressed. ^c^ Patient with treated LM (all other bars represent patients with no history of LM). NE, not evaluable.

**Extended Data Fig. 2:**
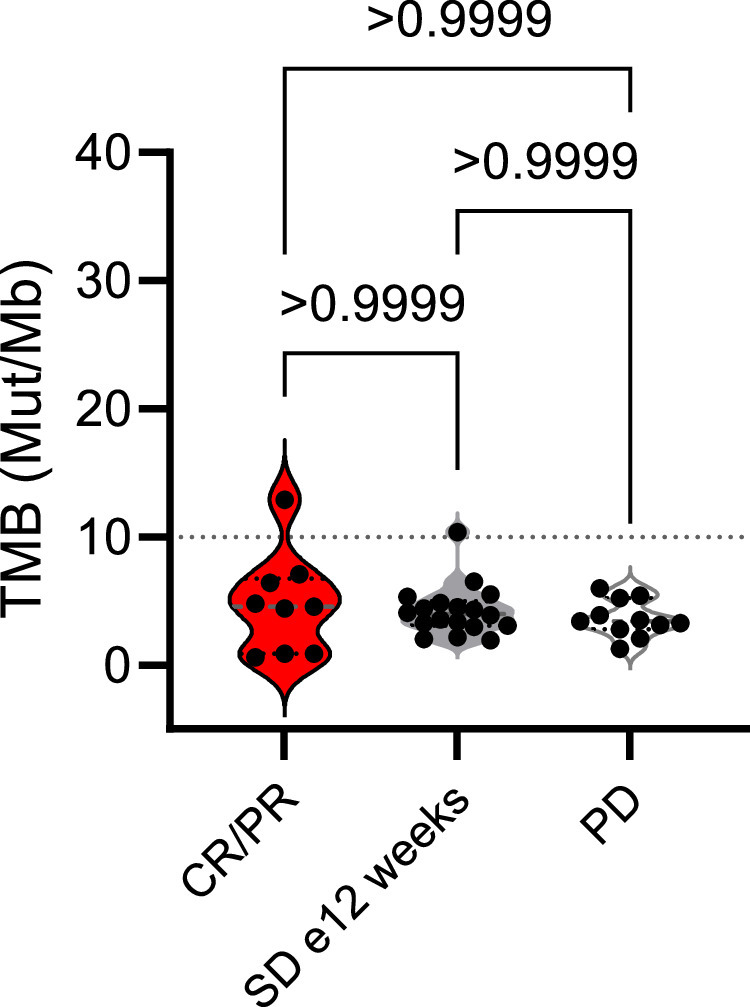
Biomarker analysis of pretreatment tumor tissue in patients with microsatellite stable metastatic colorectal cancer (*n*=38). (**a**) Clinical benefit by tumor mutational burden (TMB) at baseline (pretreatment). TMB was assessed by whole-exome sequencing (WES) using Personalis ImmunoID NeXT™ platform v1.0. TMB was defined as tumor-specific nonsynonymous somatic variants (and insertion-deletions)/megabase (Mb). Complete response (CR) or partial response (PR) and stable disease (SD) for at least 12 weeks was defined as per Response Evaluation Criteria in Solid Tumors version 1.1. Statistical analysis was performed using *Kruskal-Wallis* (ANOVA) nonparametric one-way test (*p*=0.7290, *Kruskal-Wallis* H=0.6321), followed by post-hoc *Dunn test* for adjusted multiple comparisons (CR/PR vs SD: mean rank difference= 1.111, z=0.2449, adjusted *p*=1; CR/PR vs PD: mean rank difference= 3.747, *z*=0.7503, adjusted *p*-value=1; SD vs PD: mean rank difference= 2.636, *z*=0.6199, adjusted *p*-value=1). Multiplicity adjusted *p*-values of 1 are indicated as *p*>0.9999 on the graph. Mut/Mb, mutations per megabase; PD, progressive disease.

In patients with available TMB data (*n* = 38), no significant differences were observed among responders, patients with SD or those with PD (Supplementary Fig. 1). Regarding PD-L1 expression, there are significant limitations of this dataset, making interpretation inconclusive at this time. Limitations include that PD-L1 expression was not available/assessed in all patients; biopsies were not tested consistently across sites; samples were of varying age with varying intervening treatments; and some sites were tested for PD-L1 as part of a local testing panel, whereas a subset of available samples was tested as part of the trial exploratory endpoints through a non-clinically validated, research-based PD-L1 test, 28-8, which was conducted centrally. Furthermore, there was some level of discordance between local and central testing, and the data were confounded by multiple variables. We provided response characteristics of individual responders in Fig. 4 and non-responders in Extended Data Fig. 2 and biomarker and genomics data by individual patient in Supplementary Table 7, but we have not analyzed these data due to the above-mentioned limitations.

**Extended Data Fig. 3:**
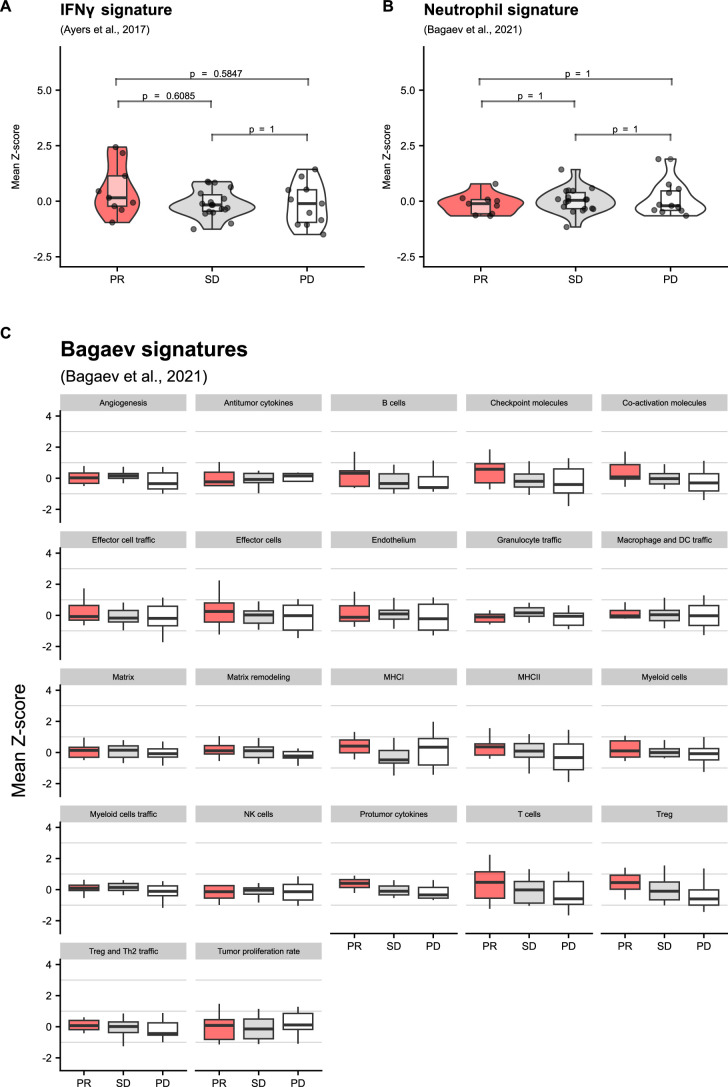
Gene expression analysis of pretreatment tumor tissue in patients with microsatellite stable metastatic colorectal cancer (*n*=38). The following plots compare patient outcomes (nine patients with partial responses [PRs], 18 with stable disease [SD] of at least 12 weeks, and 11 with progressive disease [PD]) across published gene signatures. Bulk RNA-Seq expression data was derived from pretreatment samples and the log2 gene transcripts per million (TPM) counts data were transformed to *z*-score values. The gene signatures were then calculated by averaging the gene expression values sample-wise and the results were compared across outcomes. Statistical comparisons used a two-sided nonparametric Dunn test and adjusted *p*-values were calculated with the Bonferroni method. (**a**) The interferon gamma (IFNy) signature (Ayers et al.^31^) has been used to evaluate immune checkpoint blockade therapies for treating various cancer types. The upregulation of genes in the IFNy signature are indicative of a robust immune response to cancer. (**b**) The neutrophil signature (Bagaev et al.^32^) includes a set of genes that are expressed by neutrophil cells in response to inflammation and infection. Upregulation of this signature can be used as an indicator of a poor response to checkpoint inhibitors. (**c**) The other signatures from Bagaev et al.^32^ provide additional biomarkers for probing the immune status of patients with cancer, predicting therapeutic responses, and guiding individual immunotherapy approaches. CAF, cancer-associated fibroblast; DC, dendritic cell; EMT, epithelial-to-mesenchymal transition; MHC, major histocompatibility complex; NK, natural killer; TAM, tumor-associated macrophage; T_reg_, regulatory T cells.

To determine differences in specific immune cell signatures at the RNA level at baseline (pre-treatment), RNA sequencing (RNA-seq) on bulk tumor tissue was performed, followed by determining normalized gene transcript per million counts. Counts were transformed into *z*-score values whereby gene signatures were summarized by averaging the gene expression values by sample using previously published gene signatures associated with response to ICB^31,32^. Based on low numbers of patients with available tissue for RNA-seq (nine responders; 18 with SD (≥12 weeks); and 11 with PD), statistically significant patterns were not identified among responders, those with SD or those with PD (Extended Data Fig. 3 and Supplementary Table 8)^32^.

## Discussion

MSS mCRC remains a significant clinical challenge, especially for patients who have progressed after standard chemotherapy and anti-VEGF or anti-EGFR antibodies. Available therapies have limited efficacy, and outcomes are generally poor^33–35^. To date, ICB has been ineffective in this patient population. Based on data that Fc-enhanced CTLA-4 inhibitors may overcome some mechanisms of resistance to ICB in immunologically ‘cold’ tumors, we investigated the safety and efficacy of BOT, a multifunctional Fc-enhanced CTLA-4 inhibitor, in combination with BAL, a PD-1 inhibitor, in patients with heavily pre-treated MSS mCRC. Non-clinical studies suggest that anti–CTLA-4 antibodies engineered with enhanced binding to activating FcγRs on APCs improve T cell priming and activation, along with greater T_reg_ depletion via engagement of NK cells^23,24,36^. These mechanisms may overcome the immunosuppressive TME of mCRC and drive responses even in patients with MSS and/or low TMB tumors.

In this phase 1 study including 83 patients from the dose-escalation portion of the study and 148 patients with R/R MSS mCRC treated with the combination of BOT and BAL, we showed a manageable safety profile and encouraging efficacy across tumor types. This is the first time, to our knowledge, that consistent and durable responses to ICB have been reported in difficult-to-treat patients with MSS mCRC. The safety profile of the BOT and BAL combination appears manageable and distinct from approved ICB. This is consistent with BOT’s design, which reduces complement fixation, thereby avoiding irreversible complement-mediated toxicity, such as hypophysitis^37^. Rates of high-grade treatment-related visceral toxicities outside of the gastrointestinal tract, such as hypophysitis, myocarditis, pneumonitis and hepatitis, were rare and compare favorably with other ICB combinations^38–41^. Notably, among the 148 patients with refractory CRC treated, diarrhea was the main clinically significant TRAE in 32% of patients. Based on emerging data that TNF inhibitors may allow effective management of ICB-related diarrhea/colitis, the study protocol recommended treatment with infliximab even for first-line treatment for a low-grade immune-mediated TRAE of diarrhea/colitis. Emerging evidence from clinical trials investigating CTLA-4 inhibition in lung cancer and other malignancies suggests that discontinuing therapy due to a TRAE or requiring immunosuppression to manage immune-mediated toxicities does not impact OS negatively^42–44^. Most notably, the combination of BOT and BAL resulted in promising efficacy, with an ORR of 17% (17/101 patients; ORR 18% with an additional response that was confirmed after the data cutoff) and a DCR of 61% (62/101) in patients with heavily pre-treated and refractory MSS mCRC (Figs. 2 and 3).

Patients without LM comprise approximately one-third of the mCRC population and have a better prognosis, although even these patients have limited clinical benefit with approved therapies^45–48^. The Aide et Recherche en Cancérologie Digestive Metastatic Colorectal Cancer Database comprises approximately 50 global randomized clinical trials and included about 40,000 patients with mCRC, including data from the CORRECT and RECOURSE phase 3 studies that evaluated regorafenib and trifluridine/tipiracil, respectively. In patients who received placebo, median OS in NLM patients versus those with LM was 7.9 months versus 4.1 months, and the ORR was 1.3% versus 0%. The NLM subgroup had modestly superior benefits from treatment versus the LM group (OS hazard ratio of treatment versus placebo was 0.60 versus 0.67, respectively); median OS and ORR in patients with NLM treated with trifluridine/tipiracil or regorafenib was 12.9 months (95% CI, 11.9–17.3) and 2.8% (95% CI, 1.3–5.6), respectively^49^.

As observed with other ICB agents, the presence or absence of LM may also impact the efficacy of BOT and BAL^50,51^. We prospectively enrolled patients with and without active LM to address this question and later enriched for patients with NLM with a protocol amendment. In our study, all objective responses were observed in patients with NLM (Figs. 2 and 3). There was a period during the study when, based on an amendment, only patients with no history of LM were enrolled; subsequently, enrollment was reopened to patients with treated LM as in the ongoing phase 2 study with a US Food and Drug Administration fast-track designation. Within the limitations of this smaller population of 16 patients with treated LM, outcomes appear similar to patients with no history of LM. These data provide compelling evidence that the site of metastatic disease may be an important predictive biomarker for ICB treatment efficacy. These data are consistent with additional clinical reports of activity in non-liver metastatic disease with combinations of ICB and muti-tyrosine kinase inhibitors in a variety of cancers^45,46,52,53^. Recent data suggest that the liver has a immunosuppressive environment, which may confer specific resistance of LM to ICB^47,48^. Additional studies exploring the combination of BOT plus BAL with other agents, including standard-of-care chemotherapy, such as FOLFOX and bevacizumab (NCT05608044) in the first-line setting, may improve outcomes for patients with LM by treating patients in earlier lines of therapy with higher quality T_effector_ cells and less tumoral heterogeneity.

In the context of a single-arm phase 1 study, outcomes with BOT plus BAL in a cohort of 101 response-evaluable (148 treated) patients with MSS mCRC are encouraging (Figs. 2 and 3). One limitation of this study is the lack of comparative efficacy against standard treatments for refractory MSS mCRC, such as trifluridine/tipiracil with or without bevacizumab, or regorafenib, in which response rates are known to be measurable in the low single digits^33–35^. However, most response-evaluable patients did not receive prior regorafenib (75%), trifluridine/tipiracil (86%) or both (69%), although most received prior bevacizumab (83%). Thus, the impact of prior treatments on efficacy and particularly survival remains to be elucidated. In addition, biomarker data on the study population, such as TMB, *RAS*, *BRAF* and PD-L1, were lacking in many patients. The role of PD-L1 expression in refractory MSS mCRC remains unclear but appears to have little predictive impact in first-generation ICB, and this appears to be the case with this combination, although conclusions cannot be made given the paucity of the data^54^. Another limitation of this study is that the efficacy data are still maturing, and, thus, the findings reported here may change and will be updated in a future publication.

Taken together, BOT was specifically designed to overcome the suppressive TME of immunologically ‘cold’ tumors by leveraging multiple mechanisms of action. BOT is distinct from prior drugs in the class based on its epitope binding and Fc functionality, which improve APC and NK cell binding, reduce complement fixation and enhance T_reg_ depletion. BOT represents a new generation of ICB agents capable of eliciting meaningful clinical responses in challenging ‘cold’ tumor types that have historically not responded to ICB. Furthermore, these data underscore the importance of considering the site of metastatic disease as a potential predictive biomarker for ICB efficacy, specifically the liver, in the context of MSS mCRC. This finding highlights the need to stratify patients based on the presence or absence of LM in future immunotherapy trials and to explore novel strategies to overcome the immunosuppressive liver TME. A randomized phase 2 study of this combination is underway to confirm the comparative safety and efficacy of the BOT and BAL combination (NCT05608044).

## Methods

### Study design

The C-800-01 study is an open-label, phase 1, multicenter study that evaluates the safety, tolerability and pharmacokinetic and pharmacodynamic profiles of BOT with or without BAL and assesses the maximum tolerated dose in patients with advanced solid tumors. This study aims to determine the RP2D of BOT plus BAL (NCT03860272). Patients were enrolled at 14 sites across the United States. Patients with MSS mCRC were enrolled as part of the dose-escalation portion of the study (3 + 3 design) and later as a separate indication (expansion) cohort. BOT was given Q3W or Q6W, starting at dose level 0.1 mg kg^−1^ up to 3 mg kg^−1^, administered IV for up to 2 years, or in combination with BAL 3 mg kg^−1^ Q2W administered IV for up to 2 years. A fixed-dose cohort evaluating BOT Q6W (150 mg) plus BAL Q3W (450 mg) administered IV for up to 2 years is also evaluated in this trial and is currently being evaluated in a phase 2 trial (BOT Q6W 75 mg versus 150 mg plus BAL Q2W 240 mg versus chemotherapy for refractory CRC; NCT0560844). Patients could be treated beyond progression. The C-800-01 study is currently still enrolling select indication cohorts, although the MSS mCRC expansion cohort was closed before the data cutoff. Since the original protocol (8 November 2018), there have been eight protocol amendments. The full protocol (Amendment 8) is provided as part of the Supplementary Information. A list of major changes in each amendment is summarized in Supplementary Table 9. Major changes included the addition of a safety monitoring committee (Amendment 5), the addition of a cohort to the Q6W combination arm to investigate fixed dosing (Amendment 5), an increase in the number of patients (multiple amendments) and an update of toxicity management guidelines (multiple amendments).

### Study oversight

The C-800-01 study was conducted in compliance with the Declaration of Helsinki and International Conference on Harmonization Guidelines for Good Clinical Practice and was approved by the institutional review board at each participating site: The Angeles Clinic & Research Institute, a Cedars-Sinai Affiliate; Beth Israel Deaconess Medical Center; City of Hope Comprehensive Cancer Center; Columbia University Medical Center; Dana-Farber Cancer Institute; HonorHealth Research & Innovation Institute; The University of Texas MD Anderson Cancer Center; Memorial Sloan Kettering Cancer Center; Providence Portland Cancer Center; Saint John’s Cancer Institute; University of Colorado; University of Miami Sylvester Comprehensive Cancer Center; University of Southern California Norris Comprehensive Cancer Center; and The University of Texas Health Science Center at San Antonio. All patients provided informed written consent.

### Patients

Eligible patients were age 18 years or older with measurable disease per RECIST 1.1 and an ECOG performance status of 0 or 1 with adequate end-organ function. Prior ICB was permitted. Patients must have had a confirmed diagnosis of metastatic or a locally advanced solid tumor for which no standard therapy was available or where standard therapies previously failed. Exclusion criteria included receiving any investigational agent or device at 3 weeks before the start of this study and known hypersensitivity reactions to any agents, including prior ICB and prior corticosteroid therapy, 1 week before the start of treatment. For patients with advanced CRC, tumors must not have been MSI-H/dMMR, as determined by local testing (immunohistochemistry, polymerase chain reaction or next-generation sequencing (NGS) at the discretion of the principal investigator with no protocol requirement for NGS confirmation).

### Endpoints

The primary endpoint was to assess the occurrence of any DLTs in patients in the monotherapy and combination therapy dose-escalation cohorts during the first 28 d of treatment. Secondary endpoints included the assessment of (1) the severity and duration of TEAEs and laboratory abnormalities for all dose groups (by vital signs (blood pressure, heart rate and temperature), physical examinations, 12-lead electrocardiogram, ECOG performance status and clinical laboratory assessments), according to the National Cancer Institute’s Common Terminology Criteria for Adverse Events (NCI-CTCAE) version 5.0; (2) the pharmacokinetic profile of BOT and BAL (and immunogenicity); (3) ORR; (4) DOR; (5) DCR, defined as SD or better of at least 6 weeks; and (6) PFS, all per RECIST 1.1 based on investigator assessment. Imaging was performed Q6W (±3 d) from the first treatment dose. A safety monitoring committee was established to assess safety, decide on dose escalation and define the RP2D. Exploratory endpoints included exploring biomarkers that may predict pharmacologic activity or response to BOT with or without BAL, exploring immune biomarkers in peripheral blood and tumor tissue and assessing OS (median and/or rate of OS).

### Statistical analysis

Data were evaluated as observed and are presented in a descriptive manner, with no imputation method for missing values unless otherwise specified. Descriptive statistics were used in general to summarize trial results, including means, medians, ranges and measures of variability. Qualitative variables were summarized by counts and percentages. The ORR per investigator assessment was evaluated over the entire trial period according to RECIST 1.1. For a best overall response of CR or PR, confirmation of the response was required. For patients with CR or PR, DOR was summarized using Kaplan–Meier methods, as was PFS and OS, for all patients. Median time to event(s) and 95% CI for the median were provided as required.

Exposure to BOT with or without BAL was characterized by duration (weeks), number of administrations, cumulative dose (mg kg^−1^), dose intensity (mg per kg per week), relative dose intensity (actual dose given / planned dose × 100) and number of dose delays, as a measure of compliance. Safety endpoints were tabulated by dose level, using descriptive statistics. Safety assessments were based on review of the incidence of adverse events (AEs); adverse drug reactions; immunogenicity; and changes in vital signs, electrocardiogram, body weight and laboratory values (hematology and serum chemistry).

AEs were coded according to the Medical Dictionary for Regulatory Activities (MedDRA). Severity of AEs was graded using NCI-CTCAE whenever possible. TEAEs are AEs with onset dates during the on-treatment period or the worsening of an event during the on-treatment period. Incidence of TEAEs, regardless of attribution, and TEAEs defined as related to BOT or BAL, were summarized by system organ class and preferred term and described in terms of severity and relationship to BOT or BAL. The on-treatment period was defined as time from first dose of study treatment to last dose of study treatment +30 d or the earliest date of new anti-cancer therapy −1 d, whichever occurred first. TRAEs (TEAEs related to BOT or BAL) were defined as those with onset dates or the worsening of an event during the extended on-treatment period, which was defined as time from the first dose of study treatment to last dose of study treatment +90 d or the earliest date of new anti-cancer therapy –1 d, whichever occurred first. Immune-mediated TRAEs were defined as immune-mediated TEAEs related to BOT or BAL that were treated with steroids or other immunosuppressants. All premature terminations were summarized by primary reason for study withdrawal.

The trial was conducted according to the protocol to enroll approximately 40 patients in expansion cohort 2. In an assigned dose level, patients in the dose-escalation and dose-expansion cohorts were treated in the same dose regimen and managed identically with assessments and procedures. In analyses, patients in the dose-escalation cohort are combined with the dose-expansion cohort at the same dose level, and we pooled escalation and expansion for efficacy and safety by dose.

### Genomic analysis

Genomic DNA and RNA from formalin-fixed, paraffin-embedded, tumor-containing tissue sections and matched blood samples were isolated and subjected to whole-exome paired-end sequencing and RNA-seq using the Personalis ImmunoID NeXT platform. DNA/RNA extraction, library preparation, NovaSeq (Illumina) sequencing (NovaSeq 6000 and NovaSeq X Plus sequencing platforms) and data analysis were performed at Personalis, which has a sensitivity of 99% for single-nucleotide variants (3% false-positive rate) and 94% for insertion and deletions (3% false-positive rate). The coverage was 150× for germline control blood samples and 300× for tumor samples. Somatic variants were called from the exome reads and reference human genome using hs37d5 and BWA (https://hpc.nih.gov/apps/bwa.html#:~:text=BWA%20excels%20in%20its%20speed,length%20and%20the%20maximum%20mismatches) and STAR alignment tools (https://support.illumina.com/help/BS_App_RNASeq_Alignment_OLH_1000000006112/Content/Source/Informatics/STAR_RNAseq.htm), and variants were called using MuTect (https://gatk.broadinstitute.org/hc/en-us/articles/360037593851-Mutect2), Verdict and FusionCatcher (https://github.com/ndaniel/fusioncatcher) as part of the Personalis ImmunoID NeXT analysis pipeline (https://www.personalis.com/for-biopharma/immunoid-next/). Bulk RNA-seq expression data were derived from pre-treatment samples, and log_2_ gene transcripts per million (TPM) counts were transformed to *z*-score values. Gene signatures were then calculated by averaging gene expression values sample-wise, and the results were compared across outcomes (CR or PR, SD ≥ 12 weeks or PD). R version 4.1.2 (1 November 2021) and additional R libraries (dplyr_1.1.4, ggplot2_3.5.0 and FSA_0.9.4) were used for gene expression analysis by outcome.

### Reporting summary

Further information on research design is available in the Nature Portfolio Reporting Summary linked to this article.

## Online content

Any methods, additional references, Nature Portfolio reporting summaries, source data, extended data, supplementary information, acknowledgements, peer review information; details of author contributions and competing interests; and statements of data and code availability are available at 10.1038/s41591-024-03083-7.

## Supplementary information

Supplementary Information Supplementary Data (Supplementary Tables 1–9 and Supplementary Fig. 1), Protocol Amendment 8 and Online Methods

Reporting Summary

Supplementary Table 7 Individual patient genomics and biomarker data

## Figures and Tables

**Extended Data Table 1:**
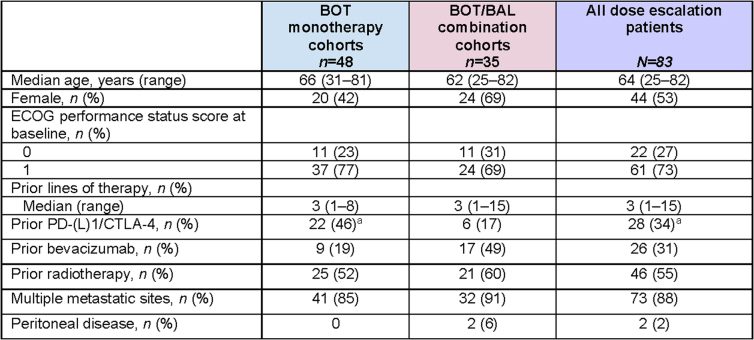
Baseline demographics and disease characteristics in all dose-escalation cohorts (*n* = 83)

^a^ One patient received prior CTLA-4 therapy in addition to prior PD-(L)1 therapy.

**Extended Data Table 2:**
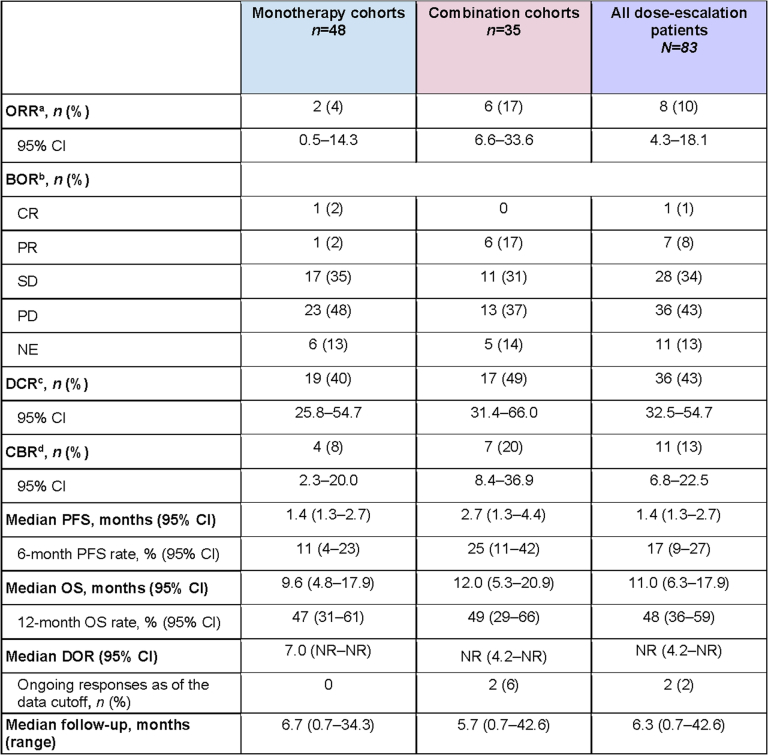
Clinical efficacy and outcomes summary in all dose-escalation cohorts (*n* = 83)

^a^ ORR is defined as the proportion of patients with a best overall response of CR or PR by RECIST 1.1.

^b^ Best overall response is defined as the best response recorded over all on-study timepoint assessments recorded before the start of a new anti-cancer therapy. Confirmed responses required confirmation after at least 28 d.

^c^ DCR is defined as proportion of patients with CR, PR or SD for at least 6 weeks.

^d^ CBR is defined as the proportion of patients with CR, PR or SD for at least 24 weeks after first dose of study drug. NE, not evaluable.

**Extended Data Table 3:**
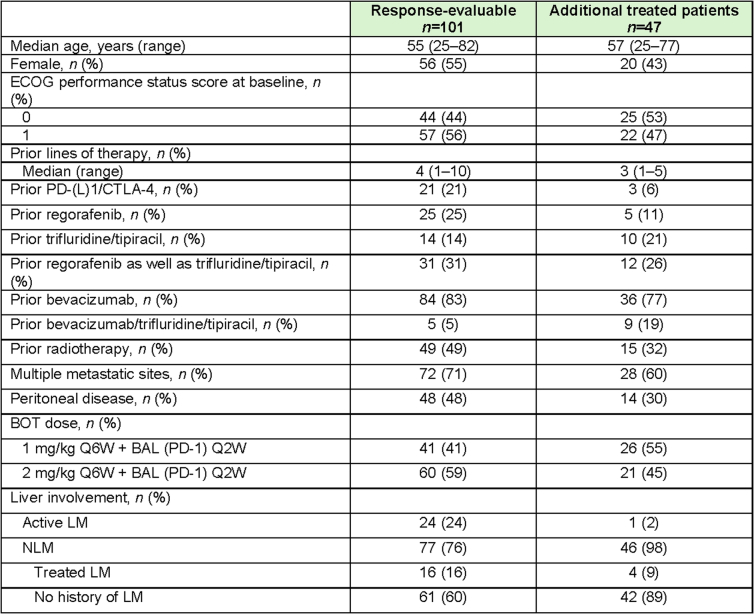
Baseline demographics and disease characteristics in response-evaluable patients (*n* = 101) and additional treated patients (*n* = 47) with MSS mCRC

## Data Availability

De-identified individual participant clinical data and WES and bulk RNA-seq data that underlie the results reported in this article are available for transfer upon request for academic use and within the limitations of the provided informed consent. Interested investigators can obtain and certify the data transfer agreement and submit requests to the corresponding author (A.B.E.-K. (elkhouei@med.usc.edu)). Investigators who consent to the terms of the data transfer agreement, including, but not limited to, the use of these data only for research purposes, and to protect the confidentiality of the data and limit the possibility of identification of patients in any way whatsoever for the duration of the agreement, will be granted access. Data will be available for request for a period of 2 years after the completion of the C-800-01 study. Requests will be evaluated on a case-by-case basis for a period of at most 2 weeks before receipt of a response.
