# Supplementary material for: Botensilimab plus balstilimab in relapsed/refractory microsatellite stable metastatic colorectal cancer: a phase 1 trial
Source: Nat Med. 2024 Jun 13;30(9):2558–67. doi: 10.1038/s41591-024-03083-7 (PMC11405281; doi:10.1038/s41591-024-03083-7)
Supplement: Supplementary file 1 — Supplementary Data (Supplementary Tables 1–9 and Supplementary Fig. 1), Protocol Amendment 8 and Online Methods [file 41591_2024_3083_MOESM1_ESM.pdf]

nature medicine

Supplementary information https://doi.org/10.1038/s41591-024-03083-7

Botensilimab plus balstilimab in relapsed/refractory microsatellite stable metastatic colorectal cancer: a phase 1 trial

In the format provided by the authors and unedited

Supplementary Data

Supplementary Table 1 I Treatment exposure in dose-escalation monotherapy cohorts (*N*=48)

	Every 3 Weeks (Q3W)						Every 6 Weeks (Q6W)			Overall Monotherapy *N*=48
	Cohort 1 (0.1 mg/kg BOT) *n*=4	Cohort 2 (0.3 mg/kg BOT) *n*=5	Cohort 3 (1.0 mg/kg BOT) *n*=8	Cohort 4 (2.0 mg/kg BOT) *n*=12	Cohort 5 (3.0 mg/kg BOT) *n*=10	Monotherapy Q3W combined *n*=39	Cohort 1 (1.0 mg/kg BOT) *n*=3	Cohort 2 (2.0 mg/kg BOT) *n*=6	Monotherapy Q6W combined *n*=9	
**Duration of BOT (days)**										
Mean (SD)	153.8 (209.4)	52.8 (45.5)	42.3 (33.4)	35.1 (51.7)	31.8 (43.6)	50.2 (80.0)	43.3 (42.0)	36.0 (49.1)	38.4 (44.3)	48.0 (74.3)
Median	75.5	49.0	34.0	22.5	22.0	23.0	44.0	22.0	43.0	23.0
Q1, Q3	32.5, 275.0	22.0, 86.0	22.0, 59.5	10.5, 26.5	1.0, 36.0	20.0, 64.0	1.0, 85.0	1.0, 43.0	1.0, 44.0	1.0, 56.5
Range	1, 463	1, 106	1, 106	1, 190	1, 149	1, 463	1, 85	1, 127	1, 127	1, 463
**Number of BOT doses received**										
Mean (SD)	8.3 (10.0)	3.4 (2.1)	2.8 (1.5)	2.3 (1.7)	2.4 (2.1)	3.2 (3.7)	2.0 (1.0)	1.8 (1.2)	1.9 (1.1)	2.9 (3.4)
Median	4.5	3.0	2.5	2.0	2.0	2.0	2.0	1.5	2.0	2.0
Q1, Q3	2.5, 14.0	2.0, 5.0	2.0, 3.0	1.5, 2.0	1.0, 2.0	2.0, 3.0	1.0, 3.0	1.0, 2.0	1.0, 2.0	1.0, 3.0
Range	1, 23	1, 6	1, 6	1, 7	1, 8	1, 23	1, 3	1, 4	1, 4	1, 23
**Dose compliance of BOT (%)**										
Mean (SD)	101.0 (2.1)	101.2 (2.3)	94.0 (15.6)	100.3 (1.9)	101.1 (1.8)	99.4 (7.4)	102.2 (2.6)	100.3 (2.5)	100.9 (2.6)	99.7 (6.8)
Median	101.2	100.0	99.4	100.7	100.2	100.2	102.8	99.45	100.0	100.2
Q1, Q3	99.6, 102.4	99.9, 100.8	97.5, 100.3	100.0, 101.3	99.8, 102.5	99.8, 101.3	99.3, 104.4	98.7, 100.2	98.9, 102.8	99.5, 101.4
Range	98.1, 103.3	99.9, 105.3	55.7, 102.0	94.9, 102.5	99.4, 104.5	55.7, 105.3	99.3, 104.4	98.7, 105.3	98.7, 105.3	55.7, 105.3

BOT, botensilimab; Q, quartile; SD, standard deviation.

Supplementary Table 2 I Treatment exposure in dose-escalation combination therapy cohorts (*N*=35)

	**Cohort 1 (0.1 mg/kg BOT Q6W + 3 mg/kg BAL Q2W) *n*=3**	**Cohort 2 (0.3 mg/kg BOT Q6W + 3mg/kg BAL Q2W) *n*=3**	**Cohort 3 (1.0 mg/kg BOT Q6W + 3mg/kg BAL Q2W) *n*=14**	**Cohort 4 (2.0 mg/kg BOT Q6W + 3mg/kg BAL Q2W) *n*=14**	**Cohort 5 (150 mg BOT Q6W + 450 mg BAL Q6W) *n*=1**	**Overall Combination *N*=35**
**Duration of BOT (days)**						
Mean (SD)	197.0 (211.4)	59.3 (22.5)	139.7 (225.9)	31.0 (44.9)	134.0 (NE)	94.1 (162.9)
Median	169.0	50.0	43.0	1.0	134.0	43.0
Q1, Q3	1.0, 421.0	43.0, 85.0	1.0, 100.0	1.0, 85.0	134.0, 134.0	1.0, 85.0
Range	29, 421	43, 85	1, 675	1, 127	134, 134	1, 675
**Number of BOT doses received**						
Mean (SD)	5.7 (5.0)	2.3 (0.6)	4.1 (5.2)	1.6 (0.9)	3.0 (NE)	3.1 (3.7)
Median	5.0	2.0	2.0	1.0	3.0	2.0
Q1, Q3	1.0, 11.0	2.0, 3.0	1.0, 3.0	1.0, 3.0	3.0, 3.0	1.0, 3.0
Range	1, 11	2, 3	1, 17	1, 3	3, 3	1, 17
**Dose compliance of BOT (%)**						
Mean (SD)	99.5 (1.0)	101.4 (1.0)	101.0 (1.9)	101.4 (2.8)	100.0 (NE)	101.0 (2.2)
Median	99.0	101.0	100.5	100.6	100.0	100.5
Q1, Q3	98.8, 100.7	100.7, 102.6	99.6, 102.3	101.0, 102.3	100.0, 100.0	99.8, 102.3
Range	98.8, 100.7	100.7, 102.6	99.4, 106.5	96.3, 107.2	100.0, 100.0	96.3, 107.2

**Duration of BAL (days)**						
Mean (SD)	206.3 (198.7)	79.0 (35.0)	157.4 (228.8)	78.5 (144.3)	134.0 (NE)	122.7 (180.1)
Median	169.0	81.0	57.0	36.0	134.0	71.0
Q1, Q3	29.0, 421.0	43.0, 113.0	29.0, 126.0	1.0, 85.0	134.0, 134.0	29.0, 113.0
Range	29, 421	43, 113	1, 675	1, 562	134, 134	1, 675
**Number of BAL doses received**						
Mean (SD)	15.7 (14.2)	6.3 (2.5)	11.1 (15.3)	5.6 (8.5)	5.0 (NE)	8.7 (11.9)
Median	13.0	6.0	5.0	3.0	5.0	4.0
Q1, Q3	3.0, 31.0	4.0, 9.0	2.0, 6.0	1.0, 6.0	5.0, 5.0	2.0, 7.0
Range	3, 31	4, 9	1, 48	1, 34	5, 5	1, 48
**Dose compliance of BAL (%)**						
Mean (SD)	100.6 (3.0)	100.4 (0.7)	101.5 (1.4)	101.9 (2.2)	100.0 (NE)	101.5 (1.8)
Median	98.9	100.5	101.3	100.9	100.0	100.9
Q1, Q3	98.8, 104.0	99.7, 101.0	100.7, 102.4	100.2, 103.2	100.0, 100.0	100.1, 102.5
Range	98.8, 104.0	99.7, 101.0	99.6, 104.8	99.6, 104.8	100.0, 100.0	98.8, 107.2
**Duration of BOT or BAL (days)**						
Mean (SD)	206.3 (198.7)	79.0 (35.0)	157.4 (228.8)	78.5 (144.3)	134.0 (NE)	122.7 (180.1)
Median	169.0	81.0	57.0	36.0	134.0	71.0
Q1, Q3	29.0, 421.0	43.0, 113.0	29.0, 126.0	1.0, 85.0	134.0, 134.0	29.0, 113.0
Range	29, 41	43, 113	1, 675	1, 562	134, 134	1, 675

**Number of BOT and BAL doses received during the same cycle**						
Mean (SD)	5.7 (5.0)	2.8 (0.6)	4.1 (5.2)	1.6 (0.9)	3.0 (NE)	3.1 (3.7)
Median	5.0	2.0	2.0	1.0	3.0	2.0
Q1, Q3	1.0, 11.0	2.0, 3.0	1.0, 3.0	1.0, 3.0	3.0, 3.0	1.0, 3.0
Range	1, 11	2, 3	1, 17	1, 3	3, 3	1, 17

BAL, balstilimab; BOT, botensilimab; NE, not evaluable; Q, quartile; Q2W, every 2 weeks; Q6W, every 6 weeks; SD, standard deviation.

Supplementary Table 3 I Summary of adverse events and listing of all treatment-related adverse events in monotherapy dose-escalation cohorts (*N*=48)

	Every 3 Weeks (Q3W)						Every 6 Weeks (Q6W)			Overall Monotherapy *N*=48
	Cohort 1 (0.1 mg/kg BOT) *n*=4	Cohort 2 (0.3 mg/kg BOT) *n*=5	Cohort 3 (1.0 mg/kg BOT) *n*=8	Cohort 4 (2.0 mg/kg BOT) *n*=12	Cohort 5 (3.0 mg/kg BOT) *n*=10	Monotherapy Q3W combined *n*=39	Cohort 1 (1.0 mg/kg BOT) *n*=3	Cohort 2 (2.0 mg/kg BOT) *n*=6	Monotherapy Q6W combined *n*=9	
**Any TEAE[a], *n* (%)**	4 (100)	5 (100)	8 (100)	12 (100)	10 (100)	39 (100)	3 (100)	5 (83)	8 (89)	47 (98)
Grade ≥3 TEAE	3 (75)	5 (100)	8 (100)	4 (33)	8 (80)	29 (72)	2 (67)	2 (33)	4 (44)	32 (67)
Serious TEAE	3 (75)	4 (80)	5 (63)	4 (33)	9 (90)	25 (64)	1 (33)	2 (33)	3 (33)	28 (58)
TEAE leading to death	2 (50)	2 (40)	2 (25)	3 (25)	1 (10)	10 (26)	0	0	0	10 (21)
TEAE leading to treatment interruption	0	1 (20)	6 (75)	4 (33)	1 (10)	12 (31)	0	1 (17)	1 (11)	13 (27)
TEAE leading to BOT discontinuation	1 (25)	3 (60)	3 (38)	5 (42)	5 (50)	17 (44)	0	1 (17)	1 (11)	18 (38)
**Any TRAE[b], *n* (%)**	1 (25)	3 (60)	8 (100)	7 (58)	10 (100)	29 (74)	3 (100)	4 (67)	7 (78)	36 (75)
Grade ≥3 TRAE	0	1 (20)	5 (63)	2 (17)	7 (70)	15 (38)	0	1 (17)	1 (11)	16 (33)
Serious TRAE	0	1 (20)	2 (25)	1 (8)	5 (50)	9 (23)	0	1 (17)	1 (11)	10 (21)
TRAE leading to death	0	0	0	0	1 (10)	1 (3)	0	0	0	1 (2)
TRAE leading to treatment interruption	0	0	5 (63)	2 (17)	1 (10)	8 (21)	0	1 (17)	1 (11)	9 (19)
TRAE leading to BOT discontinuation	0	1 (20)	2 (25)	3 (25)	4 (40)	10 (26)	0	1 (17)	1 (11)	11 (23)
**TRAE Listing by Grade**										
Grade 1	0	1 (20)	2 (25)	3 (25)	2 (20)	8 (21)	2 (67)	1 (17)	3 (33)	11 (23)

Grade 2	1 (25)	1 (20)	1 (13)	2 (17)	1 (10)	6 (15)	1 (33)	2 (33)	3 (33)	9 (19)
Grade 3	0	1 (20)	5 (63)	2 (17)	6 (60)	14 (36)	0	1 (17)	1 (11)	15 (31)
Grade 4	0	0	0	0	0	0	0	0	0	0
Grade 5	0	0	0	0	1 (10)	1 (3)	0	0	0	1 (2)
**Gastrointestinal disorders**	1 (25)	2 (40)	6 (75)	5 (42)	7 (70)	21 (54)	1 (33)	4 (67)	5 (56)	26 (54)
Diarrhea	0	1 (20)	5 (63)	3 (25)	4 (40)	13 (33)	0	1 (17)	1 (11)	14 (29)
Grade 1	0	0	3 (38)	2 (17)	2 (20)	7 (18)	0	1 (17)	1 (11)	8 (17)
Grade 2	0	1 (20)	2 (25)	0	1 (10)	4 (10)	0	0	0	4 (8)
Grade 3	0	0	0	1 (8)	1 (10)	2 (5)	0	0	0	2 (4)
Nausea	0	0	3 (38)	3 (25)	2 (20)	8 (21)	1 (33)	2 (33)	3 (33)	11 (23)
Grade 1	0	0	2 (25)	2 (17)	2 (20)	6 (15)	1 (33)	0	1 (11)	7 (15)
Grade 2	0	0	1 (13)	1 (8)	0	2 (5)	0	2 (33)	2 (22)	4 (8)
Immune-mediated enterocolitis	0	1 (20)	2 (25)	1 (8)	3 (30)	7 (18)	0	2 (33)	2 (22)	9 (19)
Grade 1	0	0	0	0	0	0	0	1 (17)	1 (11)	1 (2)
Grade 2	0	0	0	0	1 (10)	1 (3)	0	0	0	1 (2)
Grade 3	0	1 (20)	2 (25)	1 (8)	1 (10)	5 (13)	0	1 (17)	1 (11)	6 (13)
Grade 4	0	0	0	0	1 (10)	1 (3)	0	0	0	1 (2)
Colitis	0	0	0	1 (8)	2 (20)	3 (8)	0	0	0	3 (6)
Grade 2	0	0	0	1 (8)	0	1 (3)	0	0	0	1 (2)
Grade 3	0	0	0	0	2 (20)	2 (5)	0	0	0	2 (4)
Stomatitis	0	0	0	1 (8)	2 (20)	3 (8)	0	0	0	3 (6)

Grade 1	0	0	0	0	1 (10)	1 (3)	0	0	0	1 (2)
Grade 2	0	0	0	0	1 (10)	1 (3)	0	0	0	1 (2)
Grade 3	0	0	0	1 (8)	0	1 (3)	0	0	0	1 (2)
Abdominal pain	0	0	1 (13)	0	1 (10)	2 (5)	0	0	0	2 (4)
Grade 1	0	0	1 (13)	0	0	1 (3)	0	0	0	1 (2)
Grade 2	0	0	0	0	1 (10)	1 (3)	0	0	0	1 (2)
Constipation	0	0	0	1 (8)	0	1 (3)	0	0	0	1 (2)
Grade 1	0	0	0	1 (8)	0	1 (3)	0	0	0	1 (2)
Flatulence	1 (25)	0	0	0	0	1 (3)	0	0	0	1 (2)
Grade 1	1 (25)	0	0	0	0	1 (3)	0	0	0	1 (2)
Large intestine perforation	0	0	0	0	1 (10)	1 (3)	0	0	0	1 (2)
Grade 5	0	0	0	0	1 (10)	1 (3)	0	0	0	1 (2)
**General disorders and administration site conditions**	1 (25)	3 (60)	3 (38)	2 (17)	5 (50)	14 (36)	1 (33)	3 (50)	4 (44)	18 (38)
Fatigue	1 (25)	1 (20)	3 (38)	2 (17)	2 (20)	9 (23)	1 (33)	3 (50)	4 (44)	13 (27)
Grade 1	1 (25)	1 (20)	0	0	0	2 (5)	1 (33)	2 (33)	3 (33)	5 (10)
Grade 2	0	0	2 (25)	2 (17)	2 (20)	6 (15)	0	1 (17)	1 (11)	7 (15)
Grade 3	0	0	1 (13)	0	0	1 (3)	0	0	0	1 (2)
Pyrexia	1 (25)	1 (20)	0	0	2 (20)	4 (10)	0	0	0	4 (8)
Grade 1	1 (25)	1 (20)	0	0	2 (20)	4 (10)	0	0	0	4 (8)
Asthenia	0	1 (20)	0	1 (8)	0	2 (5)	0	0	0	2 (4)
Grade 1	0	0	0	1 (8)	0	1 (3)	0	0	0	1 (2)

Grade 3	0	1 (20)	0	0	0	1 (3)	0	0	0	1 (2)
Chills	0	0	0	0	1 (10)	1 (3)	0	1 (17)	1 (11)	2 (4)
Grade 1	0	0	0	0	1 (10)	1 (3)	0	1 (17)	1 (11)	2 (4)
**Skin and subcutaneous tissue disorders**	1 (25)	1 (20)	3 (38)	4 (33)	4 (40)	13 (33)	0	4 (67)	4 (44)	17 (35)
Pruritis	1 (25)	0	1 (13)	3 (25)	2 (20)	7 (18)	0	1 (17)	2 (22)	9 (19)
Grade 1	1 (25)	0	0	2 (17)	1 (10)	4 (10)	0	1 (17)	1 (11)	5 (10)
Grade 2	0	0	1 (13)	1 (8)	1 (10)	3 (8)	0	1 (17)	1 (11)	4 (8)
Rash maculopapular	0	0	1 (13)	0	2 (20)	3 (8)	0	1 (17)	1 (11)	4 (8)
Grade 1	0	0	0	0	1 (10)	1 (3)	0	0	0	1 (2)
Grade 2	0	0	1 (13)	0	0	1 (3)	0	1 (17)	1 (11)	2 (4)
Grade 3	0	0	0	0	1 (10)	1 (3)	0	0	0	1 (2)
Rash	0	0	1 (13)	1 (8)	0	2 (5)	0	1 (17)	1 (11)	3 (6)
Grade 1	0	0	0	1 (8)	0	1 (3)	0	1 (17)	1 (11)	2 (4)
Grade 3	0	0	1 (13)	0	0	1 (3)	0	0	0	1 (2)
Dry skin	0	0	0	0	1 (10)	1 (3)	0	0	0	1 (2)
Grade 1	0	0	0	0	1 (10)	1 (3)	0	0	0	1 (2)
Eczema	0	0	1 (13)	0	0	1 (3)	0	0	0	1 (2)
Grade 3	0	0	1 (13)	0	0	1 (3)	0	0	0	1 (2)
Night sweats	0	1 (20)	0	0	0	1 (3)	0	0	0	1 (2)
Grade 1	0	1 (20)	0	0	0	1 (3)	0	0	0	1 (2)
Palmar erythema	1 (25)	0	0	0	0	1 (3)	0	0	0	1 (2)

Grade 1	1 (25)	0	0	0	0	1 (3)	0	0	0	1 (2)
Photosensitivity reaction	1 (25)	0	0	0	0	1 (3)	0	0	0	1 (2)
Grade 1	1 (25)	0	0	0	0	1 (3)	0	0	0	1 (2)
Pruritic rash	0	0	0	1 (8)	0	1 (3)	0	0	0	1 (2)
Grade 2	0	0	0	1 (8)	0	1 (3)	0	0	0	1 (2)
Skin ulcer	0	0	1 (13)	0	0	1 (3)	0	0	0	1 (2)
Grade 3	0	0	1 (13)	0	0	1 (3)	0	0	0	1 (2)
**Metabolism and nutrition disorders**	0	1 (20)	2 (25)	1 (17)	2 (20)	7 (18)	0	1 (17)	1 (11)	8 (17)
Decreased appetite	0	0	2 (25)	2 (17)	2 (20)	6 (15)	0	1 (17)	1 (11)	7 (15)
Grade 1	0	0	0	2 (17)	1 (10)	3 (8)	0	1 (17)	1 (11)	4 (8)
Grade 2	0	0	2 (25)	0	0	2 (5)	0	0	0	2 (4)
Grade 3	0	0	0	0	1 (10)	1 (3)	0	0	0	1 (2)
Dehydration	0	0	1 (13)	1 (8)	1 (10)	3 (8)	0	0	0	3 (6)
Grade 2	0	0	1 (13)	1 (8)	1 (10)	3 (8)	0	0	0	3 (6)
Hypomagnesemia	0	1 (20)	0	1 (8)	0	2 (5)	0	0	0	2 (4)
Grade 1	0	1 (20)	0	1 (8)	0	2 (5)	0	0	0	2 (4)
Alkalosis	0	0	1 (13)	0	0	1 (3)	0	0	0	1 (2)
Grade 1	0	0	1 (13)	0	0	1 (3)	0	0	0	1 (2)
Hypokalemia	0	0	0	1 (8)	0	1 (3)	0	0	0	1 (2)
Grade 1	0	0	0	1 (8)	0	1 (3)	0	0	0	1 (2)
**Nervous system disorders**	0	0	0	3 (25)	2 (20)	5 (13)	0	0	0	5 (10)

Dizziness	0	0	0	2 (17)	0	2 (5)	0	0	0	2 (4)
Grade 1	0	0	0	2 (17)	0	2 (5)	0	0	0	2 (4)
Dysgeusia	0	0	0	0	1 (10)	1 (3)	0	0	0	1 (2)
Grade 1	0	0	0	0	1 (10)	1 (3)	0	0	0	1 (2)
Headache	0	0	0	1 (8)	0	1 (3)	0	0	0	1 (2)
Grade 1	0	0	0	1 (8)	0	1 (3)	0	0	0	1 (2)
Lethargy	0	0	0	0	1 (10)	1 (3)	0	0	0	1 (2)
Grade 1	0	0	0	0	1 (10)	1 (3)	0	0	0	1 (2)
**Endocrine disorders**	1 (25)	0	0	1 (8)	0	2 (5)	1 (33)	1 (17)	2 (22)	4 (8)
Hyperthyroidism	1 (25)	0	0	1 (8)	0	2 (5)	1 (33)	0	1 (11)	3 (6)
Grade 1	0	0	0	1 (8)	0	1 (3)	0	0	0	1 (2)
Grade 2	1 (25)	0	0	0	0	1 (3)	1 (33)	0	1 (11)	2 (4)
Hypothyroidism	1 (25)	0	0	0	0	1 (3)	0	1 (17)	1 (11)	2 (4)
Grade 1	0	0	0	0	0	0	0	1 (17)	1 (11)	1 (2)
Grade 2	1 (25)	0	0	0	0	1 (3)	0	0	0	1 (2)
**Investigations**	1 (25)	0	0	1 (8)	1 (10)	3 (8)	0	1 (17)	1 (11)	4 (8)
Blood creatinine increased	0	0	0	1 (8)	0	1 (3)	0	1 (17)	1 (11)	2 (4)
Grade 2	0	0	0	1 (8)	0	1 (3)	0	1 (17)	1 (11)	2 (4)
Alanine aminotransferase increased	0	0	0	0	1 (10)	1 (3)	0	0	0	1 (2)
Grade 1	0	0	0	0	1 (10)	1 (3)	0	0	0	1 (2)
Blood thyroid stimulating hormone increased	1 (25)	0	0	0	0	1 (3)	0	0	0	1 (2)

Grade 1	1 (25)	0	0	0	0	1 (3)	0	0	0	1 (2)
**Injury, poisoning and procedural complications**	0	1 (20)	1 (13)	0	1 (10)	3 (8)	0	0	0	3 (6)
Infusion-related reaction	0	1 (20)	1 (13)	0	1 (10)	3 (8)	0	0	0	3 (6)
Grade 1	0	1 (20)	0	0	0	1 (3)	0	0	0	1 (2)
Grade 2	0	0	1 (13)	0	0	1 (3)	0	0	0	1 (2)
Grade 3	0	0	0	0	1 (10)	1 (3)	0	0	0	1 (2)
**Musculoskeletal and connective tissue disorders**	0	0	1 (13)	1 (8)	0	2 (5)	1 (33)	0	1 (11)	3 (6)
Myalgia	0	0	1 (13)	1 (8)	0	2 (5)	1 (33)	0	1 (11)	3 (6)
Grade 1	0	0	1 (13)	1 (8)	0	2 (5)	1 (33)	0	1 (11)	3 (6)
Arthralgia	0	0	1 (13)	1 (8)	0	2 (5)	0	0	0	2 (4)
Grade 1	0	0	1 (13)	1 (8)	0	2 (5)	0	0	0	2 (4)
**Blood and lymphatic system disorders**	0	0	1 (13)	0	1 (10)	2 (5)	0	0	0	2 (4)
Anemia	0	0	1 (13)	0	1 (10)	2 (5)	0	0	0	2 (4)
Grade 1	0	0	1 (13)	0	0	1 (3)	0	0	0	1 (2)
Grade 2	0	0	0	0	1 (10)	1 (3)	0	0	0	1 (2)
**Respiratory, thoracic and mediastinal disorders**	0	0	1 (13)	0	1 (10)	2 (5)	0	0	0	2 (4)
Cough	0	0	1 (13)	0	0	1 (3)	0	0	0	1 (2)
Grade 1	0	0	1 (13)	0	0	1 (3)	0	0	0	1 (2)
Dysphonia	0	0	0	0	1 (10)	1 (3)	0	0	0	1 (2)
Grade 2	0	0	0	0	1 (10)	1 (3)	0	0	0	1 (2)

**Vascular disorders**	0	0	2 (25)	0	0	2 (5)	0	0	0	2 (4)
Hypotension	0	0	2 (25)	0	0	2 (5)	0	0	0	2 (4)
Grade 1	0	0	1 (13)	0	0	1 (3)	0	0	0	1 (2)
Grade 2	0	0	1 (13)	0	0	1 (3)	0	0	0	1 (2)
**Eye disorders**	0	0	0	0	0	0	0	1 (17)	1 (11)	1 (2)
Vision blurred	0	0	0	0	0	0	0	1 (17)	1 (11)	1 (2)
Grade 1	0	0	0	0	0	0	0	1 (17)	1 (11)	1 (2)
**Infections and infestations**	0	1 (20)	0	0	0	1 (3)	0	0	0	1 (2)
Sinusitis	0	1 (20)	0	0	0	1 (3)	0	0	0	1 (2)
Grade 2	0	1 (20)	0	0	0	1 (3)	0	0	0	1 (2)
**Benign neoplasms, malignant, and unspecified (including cysts and polyps)**	0	0	0	0	1 (10)	1 (3)	0	0	0	1 (2)
Tumor pain	0	0	0	0	1 (10)	1 (3)	0	0	0	1 (2)
Grade 3	0	0	0	0	1 (10)	1 (3)	0	0	0	1 (2)

[a]Treatment-emergent adverse events (TEAEs) were coded using the Medical Dictionary Regulatory Activities, Version 22.1, classified by system organ class and preferred term. [b]Treatment-emergent adverse events related to botensilimab (BOT) or balstilimab (BAL) were coded using the Medical Dictionary Regulatory Activities, Version 22.1, classified by system organ class and preferred term. Treatment-related adverse events (TRAEs; TEAEs related to BOT or BAL) were defined as adverse events with onset dates or the worsening of an event during the extended on-treatment period, which was defined as time from the first dose of study treatment to last dose of study treatment +90 days, or the earliest date of new anticancer therapy –1 day, whichever occurred first.

BOT, botensilimab.

Supplementary Table 4 I Summary of adverse events and listing of all treatment-related adverse events in combination therapy dose-escalation cohorts (*N*=35)

	Cohort 1 (0.1 mg/kg BOT Q6W + 3 mg/kg BAL Q2W) *n*=3	Cohort 2 (0.3 mg/kg BOT Q6W + 3mg/kg BAL Q2W) *n*=3	Cohort 3 (1.0 mg/kg BOT Q6W + 3mg/kg BAL Q2W) *n*=14	Cohort 4 (2.0 mg/kg BOT Q6W + 3mg/kg BAL Q2W) *n*=14	Cohort 5 (150 mg BOT Q6W + 450 mg BAL Q6W) *n*=1	Overall Combination *N*=35
**Any TEAE [a], *n* (%)**	3 (100)	3 (100)	14 (100)	14 (100)	1 (100)	35 (100)
Grade ≥3 TEAE	1 (33)	2 (67)	10 (71)	10 (71)	1 (100)	24 (69)
Serious TEAE	1 (33)	2 (67)	8 (57)	11 (79)	1 (100)	23 (66)
TEAE leading to death	0	2 (67)	1 (7)	5 (36)	1 (100)	13 (37)
TEAE leading to treatment interruption	0	0	5 (36)	7 (50)	1 (100)	13 (37)
TEAE leading to treatment discontinuation	0	0	6 (43)	7 (50)	0	13 (37)
TEAE leading to BOT discontinuation	0	0	6 (43)	5 (36)	0	11 (31)
TEAE leading to BAL discontinuation	0	0	6 (43)	6 (43)	0	12 (34)
TEAE leading to BOT and BAL discontinuation	0	0	5 (36)	4 (29)	0	9 (26)
**Any TRAE[b], *n* (%)**	2 (67)	2 (67)	13 (93)	11 (79)	1 (100)	29 (83)
Grade ≥3 TRAE	0	0	5 (36)	3 (21)	1 (100)	9 (26)
Serious TRAE	0	0	6 (43)	6 (43)	0	12 (34)
TRAE leading to death	0	0	0	0	0	0
TRAE leading to treatment interruption	0	0	4 (29)	6 (43)	1 (100)	11 (31)
TRAE leading to treatment discontinuation	0	0	5 (36)	4 (29)	0	9 (26)

TRAE leading to BOT discontinuation	0	0	5 (36)	3 (21)	0	8 (23)
TRAE leading to BAL discontinuation	0	0	5 (36)	3 (21)	0	8 (23)
TRAE leading to BOT and BAL discontinuation	0	0	4 (29)	2 (14)	0	6 (17)
**TRAE Listing by Grade**						
Grade 1	1 (33)	1 (33)	4 (29)	3 (21)	0	9 (26)
Grade 2	1 (33)	1 (33)	4 (29)	5 (36)	0	11 (31)
Grade 3	0	0	4 (29)	3 (21)	1 (100)	8 (23)
Grade 4	0	0	1 (7)	0	0	1 (3)
Grade 5	0	0	0	0	0	0
**Gastrointestinal disorders**	0	0	9 (64)	7 (50)	1 (100)	17 (49)
Diarrhea	0	0	5 (36)	4 (29)	0	9 (26)
Grade 1	0	0	3 (21)	0	0	3 (9)
Grade 2	0	0	1 (7)	4 (29)	0	5 (14)
Grade 3	0	0	1 (7)	0	0	1 (3)
Immune-mediated enterocolitis	0	0	2 (14)	4 (29)	0	6 (17)
Grade 2	0	0	0	3 (21)	0	3 (9)
Grade 3	0	0	2 (14)	1 (7)	0	3 (9)
Nausea	0	0	4 (29)	1 (7)	0	5 (14)
Grade 1	0	0	1 (7)	0	0	1 (3)
Grade 2	0	0	3 (21)	1 (7)	0	4 (11)

Stomatitis	0	0	3 (21)	1 (7)	0	4 (11)
Grade 1	0	0	1 (7)	0	0	1 (3)
Grade 2	0	0	1 (7)	1 (7)	0	2 (6)
Grade 3	0	0	1 (7)	0	0	1 (3)
Vomiting	0	0	2 (14)	1 (7)	0	3 (9)
Grade 1	0	0	1 (7)	0	0	1 (3)
Grade 2	0	0	1 (7)	1 (7)	0	2 (6)
Abdominal pain	0	0	2 (14)	0	0	2 (6)
Grade 1	0	0	2 (14)	0	0	2 (6)
Abdominal distension	0	0	0	1 (7)	0	1 (3)
Grade 1	0	0	0	1 (7)	0	1 (3)
Colitis	0	0	1 (7)	0	0	1 (3)
Grade 2	0	0	1 (7)	0	0	1 (3)
Enterocolitis	0	0	0	1 (7)	0	1 (3)
Grade 3	0	0	0	1 (7)	0	1 (3)
Soft feces	0	0	0	0	1 (100)	1 (3)
Grade 1	0	0	0	0	1 (100)	1 (3)
**General disorders and administration site conditions**	1 (33)	0	8 (57)	4 (29)	0	13 (37)
Fatigue	1 (33)	0	6 (43)	3 (21)	0	10 (29)
Grade 1	1 (33)	0	1 (7)	0	0	2 (6)
Grade 2	0	0	4 (29)	3 (21)	0	7 (20)

Grade 3	0	0	1 (7)	1 (7)	0	2 (6)
Influenza-like illness	0	0	1 (7)	1 (7)	0	2 (6)
Grade 1	0	0	1 (7)	1 (7)	0	2 (6)
Pain	0	0	2 (14)	0	0	2 (6)
Grade 1	0	0	2 (14)	0	0	2 (6)
Pyrexia	0	0	1 (7)	1 (7)	0	2 (6)
Grade 1	0	0	1 (7)	1 (7)	0	2 (6)
**Skin and subcutaneous tissue disorders**	0	1 (33)	6 (43)	6 (43)	0	13 (37)
Pruritis	0	1 (33)	2 (14)	3 (21)	0	6 (17)
Grade 1	0	1 (33)	1 (7)	2 (14)	0	4 (11)
Grade 2	0	0	1 (7)	1 (7)	0	2 (6)
Rash maculopapular	0	0	3 (21)	2 (14)	0	5 (14)
Grade 1	0	0	1 (7)	1 (7)	0	2 (6)
Grade 2	0	0	2 (14)	1 (7)	0	3 (9)
Rash	0	0	1 (7)	2 (14)	0	3 (9)
Grade 1	0	0	1 (7)	1 (7)	0	2 (6)
Grade 2	0	0	0	1 (7)	0	1 (3)
Immune-mediated dermatitis	0	0	1 (7)	1 (7)	0	2 (6)
Grade 1	0	0	1 (7)	1 (7)	0	2 (6)
Palmar-plantar erythrodysesthesia syndrome	0	0	1 (7)	1 (7)	0	2 (6)

Grade 1	0	0	1 (7)	0	0	1 (3)
Grade 2	0	0	0	1 (7)	0	1 (3)
Dermatitis acneiform	0	0	1 (7)	0	0	1 (3)
Grade 1	0	0	1 (7)	0	0	1 (3)
**Metabolism and nutrition disorders**	0	1 (33)	4 (29)	5 (36)	1 (100)	11 (31)
Decreased appetite	0	0	4 (29)	2 (14)	1 (100)	7 (20)
Grade 1	0	0	1 (7)	1 (7)	1 (100)	3 (9)
Grade 2	0	0	3 (21)	1 (7)	0	4 (11)
Hypoalbuminemia	0	1 (33)	0	1 (7)	0	2 (6)
Grade 1	0	0	0	1 (7)	0	1 (3)
Grade 2	0	1 (33)	0	0	0	1 (3)
Hyperkalemia	0	0	0	1 (7)	0	1 (3)
Grade 2	0	0	0	1 (7)	0	1 (3)
Hypocalcemia	0	0	0	1 (7)	0	1 (3)
Grade 1	0	0	0	1 (7)	0	1 (3)
Hypokalemia	0	0	0	1 (7)	0	1 (3)
Grade 2	0	0	0	1 (7)	0	1 (3)
Hypomagnesemia	0	0	0	1 (7)	0	1 (3)
Grade 1	0	0	0	1 (7)	0	1 (3)
**Investigations**	0	0	4 (29)	4 (29)	1 (100)	9 (26)
Aspartate aminotransferase increased	0	0	1 (7)	2 (14)	1 (100)	4 (11)

Grade 1	0	0	1 (7)	2 (14)	0	3 (9)
Grade 2	0	0	0	0	1 (100)	1 (3)
Alanine aminotransferase increased	0	0	1 (7)	1 (7)	1 (100)	3 (9)
Grade 1	0	0	1 (7)	1 (7)	0	2 (6)
Grade 3	0	0	0	0	1 (100)	1 (3)
Blood alkaline phosphatase increased	0	0	1 (7)	0	1 (100)	2 (6)
Grade 2	0	0	1 (7)	0	0	1 (3)
Grade 3	0	0	0	0	1 (100)	1 (3)
Blood creatinine increased	0	0	1 (7)	1 (7)	0	2 (6)
Grade 1	0	0	1 (7)	0	0	1 (3)
Grade 2	0	0	0	1 (7)	0	1 (3)
Weight decreased	0	0	2 (14)	0	0	2 (6)
Grade 2	0	0	2 (14)	0	0	2 (6)
Blood bilirubin increased	0	0	0	0	1 (100)	1 (3)
Grade 2	0	0	0	0	1 (100)	1 (3)
Platelet count decreased	0	0	0	1 (7)	0	1 (3)
Grade 2	0	0	0	1 (7)	0	1 (3)
White blood cell count decreased	0	0	0	1 (7)	0	1 (3)
Grade 1	0	0	0	1 (7)	0	1 (3)
**Musculoskeletal and connective tissue disorders**	1 (33)	0	5 (36)	3 (21)	0	9 (26)

Arthralgia	0	0	1 (7)	2 (14)	0	3 (9)
Grade 1	0	0	1 (7)	1 (7)	0	2 (6)
Grade 2	0	0	0	1 (7)	0	1 (3)
Myalgia	0	0	2 (14)	0	0	2 (6)
Grade 1	0	0	2 (14)	0	0	2 (6)
Polyarthritis	1 (33)	0	1 (7)	0	0	2 (6)
Grade 1	1 (33)	0	0	0	0	1 (3)
Grade 3	0	0	1 (7)	0	0	1 (3)
Muscular weakness	0	0	0	1 (7)	0	1 (3)
Grade 1	0	0	0	1 (7)	0	1 (3)
Pain in extremity	0	0	1 (7)	0	0	1 (3)
Grade 1	0	0	1 (7)	0	0	1 (3)
**Endocrine disorders**	1 (33)	0	5 (36)	2 (14)	0	8 (23)
Adrenal insufficiency	0	0	2 (14)	1 (7)	0	3 (9)
Grade 2	0	0	2 (14)	1 (7)	0	3 (9)
Hyperthyroidism	0	0	2 (14)	0	0	2 (6)
Grade 1	0	0	1 (7)	0	0	1 (3)
Grade 2	0	0	1 (7)	0	0	1 (3)
Hypothyroidism	1 (33)	0	0	0	0	1 (3)
Grade 2	1 (33)	0	0	0	0	1 (3)
Immune-mediated hypothyroidism	0	0	1 (7)	0	0	1 (3)

Grade 2	0	0	1 (7)	0	0	1 (3)
Immune-mediated thyroiditis	0	0	0	1 (7)	0	1 (3)
Grade 1	0	0	0	1 (7)	0	1 (3)
**Nervous system disorders**	0	0	4 (29)	2 (14)	0	6 (17)
Headache	0	0	2 (14)	1 (7)	0	3 (9)
Grade 1	0	0	2 (14)	1 (7)	0	3 (9)
Dizziness	0	0	1 (7)	1 (7)	0	2 (6)
Grade 1	0	0	0	1 (7)	0	1 (3)
Grade 2	0	0	1 (7)	0	0	1 (3)
Peripheral sensory neuropathy	0	0	1 (7)	0	0	1 (3)
Grade 1	0	0	1 (7)	0	0	1 (3)
**Respiratory, thoracic and mediastinal disorders**	0	0	1 (7)	1 (7)	1 (100)	3 (9)
Dyspnea	0	0	1 (7)	0	1 (100)	2 (6)
Grade 1	0	0	1 (7)	0	0	1 (3)
Grade 2	0	0	0	0	1 (100)	1 (3)
Acute respiratory distress syndrome	0	0	0	1 (7)	0	1 (3)
Grade 2	0	0	0	1 (7)	0	1 (3)
Cough	0	0	0	0	1 (100)	1 (3)
Grade 2	0	0	0	0	1 (100)	1 (3)
**Blood and lymphatic system disorders**	0	0	1 (7)	0	0	1 (3)

Thrombocytopenia	0	0	1 (7)	0	0	1 (3)
Grade 4	0	0	1 (7)	0	0	1 (3)
**Eye disorders**	0	0	1 (7)	0	0	1 (3)
Vision blurred	0	0	1 (7)	0	0	1 (3)
Grade 2	0	0	1 (7)	0	0	1 (3)
**Infections and infestations**	0	0	0	1 (7)	0	1 (3)
Infection	0	0	0	1 (7)	0	1 (3)
Grade 2	0	0	0	1 (7)	0	1 (3)
**Renal and urinary disorders**	0	0	0	1 (7)	0	1 (3)
Acute kidney injury	0	0	0	1 (7)	0	1 (3)
Grade 3	0	0	0	1 (7)	0	1 (3)

[a]Treatment-emergent adverse events (TEAEs) were coded using the Medical Dictionary Regulatory Activities, Version 22.1, classified by system organ class and preferred term. [b]Treatment-emergent adverse events (TEAE) related to botensilimab (BOT) or balstilimab (BAL) were coded using the Medical Dictionary Regulatory Activities, Version 22.1, classified by system organ class and preferred term. Treatment-related adverse events (TRAEs; TEAEs related to BOT or BAL) were defined as adverse events with onset dates or the worsening of an event during the extended on-treatment period, which was defined as time from the first dose of study treatment to last dose of study treatment +90 days, or the earliest date of new anticancer therapy –1 day, whichever occurred first.

BAL, balstilimab; BOT, botensilimab.

Supplementary Table 5 | Summary of adverse events and listing of all treatment-related adverse events in all treated patients with microsatellite stable metastatic colorectal cancer (*N*=148)

	**All treated** **_N_=148**
**Any TEAE [a], *n* (%)**	148 (100)
Grade ≥3 TEAE	90 (61)
Serious TEAE	86 (58)
TEAE leading to death	11 (7)
TEAE leading to treatment interruption	81 (55)
TEAE leading to treatment discontinuation	50 (34)
TEAE leading to BOT discontinuation	50 (34)
TEAE leading to BAL discontinuation	29 (20)
TEAE leading to BOT and BAL discontinuation	26 (18)
**Any TRAE[b], *n* (%)**	131 (89)
Grade ≥3 TRAE	48 (32)
Serious TRAE	52 (35)
TRAE leading to death	0
TRAE leading to treatment interruption	47 (32)
TRAE leading to treatment discontinuation	42 (28)
TRAE leading to BOT discontinuation	42 (28)
TRAE leading to BAL discontinuation	21 (14)
TRAE leading to BOT and BAL discontinuation	18 (12)

TRAE Listing by Grade
***N*=148**

	**Grade 1**	**Grade 2**	**Grade 3**	**Grade 4**	**All-grade**
**Any TRAE[a], *n* (%)**	27 (18)	56 (38)	46 (31)	2 (1)	131 (89)
Fatigue	27 (18)	23 (16)	2 (1)	0	52 (35)
Diarrhea	18 (12)	21 (14)	8 (5)	0	47 (32)
Pyrexia	17 (11)	15 (10)	4 (3)	0	36 (24)
Decreased appetite	21 (14)	12 (8)	0	0	33 (22)
Chills	29 (20)	1 (<1)	0	0	30 (20)
Pruritis	21 (14)	7 (5)	0	0	28 (19)
Nausea	20 (14)	5 (3)	2 (1)	0	27 (18)
Colitis	3 (2)	18 (12)	4 (3)	1 (<1)	26 (18)
Rash maculo-papular	18 (12)	7 (5)	0	0	25 (17)
Arthralgia	16 (11)	5 (3)	0	0	21 (14)
Alanine aminotransferase increased	11 (7)	6 (4)	2 (1)	0	19 (13)
Anemia	8 (5)	8 (5)	0	0	16 (11)
Headache	14 (9)	1 (<1)	1 (<1)	0	16 (11)
Myalgia	10 (7)	6 (4)	0	0	16 (11)
Immune-mediated enterocolitis	1 (<1)	7 (5)	6 (4)	0	14 (9)
Rash	10 (7)	4 (3)	0	0	14 (9)
Vomiting	9 (6)	4 (3)	1 (<1)	0	14 (9)

Aspartate aminotransferase increased	8 (5)	3 (2)	2 (1)	0	13 (9)
Hyperhidrosis	10 (7)	1 (<1)	0	0	11 (7)
Stomatitis	5 (3)	5 (3)	0	0	10 (7)
Blood alkaline phosphatase increased	6 (4)	2 (1)	0	0	8 (5)
Dyspnea	4 (3)	4 (3)	0	0	8 (5)
Blood creatine phosphokinase increased	2 (1)	0	4 (3)	1 (<1)	7 (5)
Blood creatinine increased	4 (3)	3 (2)	0	0	7 (5)
Dizziness	6 (4)	1 (<1)	0	0	7 (5)
Dry mouth	7 (5)	0	0	0	7 (5)
Hypothyroidism	1 (<1)	6 (4)	0	0	7 (5)
Abdominal pain	4 (3)	2 (1)	0	0	6 (4)
Blood thyroid stimulating hormone increased	5 (3)	1 (<1)	0	0	6 (4)
Cough	4 (3)	2 (1)	0	0	6 (4)
Hyperthyroidism	6 (4)	0	0	0	6 (4)
Neutrophil count decreased	3 (2)	2 (1)	1 (<1)	0	6 (4)
Adrenal insufficiency	1 (<1)	2 (1)	2 (1)	0	5 (3)
Hypokalemia	3 (2)	2 (1)	0	0	5 (3)
Influenza-like illness	5 (3)	0	0	0	5 (3)
Lymphocyte count decreased	0	4 (3)	1 (<1)	0	5 (3)
Malaise	3 (2)	2 (1)	0	0	5 (3)
Weight decreased	4 (3)	1 (<1)	0	0	5 (3)
Dehydration	0	3 (2)	1 (<1)	0	4 (3)
Dermatitis acneiform	4 (3)	0	0	0	4 (3)
Acute kidney injury	0	0	3 (2)	0	3 (2)
Amylase increased	1 (<1)	1 (<1)	1 (<1)	0	3 (2)
Conjunctivitis	2 (1)	1 (<1)	0	0	3 (2)
Dry skin	3 (2)	0	0	0	3 (2)
Hypomagnesemia	3 (2)	0	0	0	3 (2)
Immune-mediated dermatitis	1 (<1)	2 (1)	0	0	3 (2)
Immune-mediated lung disease	0	2 (1)	1 (<1)	0	3 (2)
Infusion-related reaction	2 (1)	1 (<1)	0	0	3 (2)
Lipase increased	0	2 (1)	1 (<1)	0	3 (2)
Oropharyngeal pain	3 (2)	0	0	0	3 (2)
Pneumonitis	0	2 (1)	1 (<1)	0	3 (2)
White blood cell count decreased	2 (1)	1 (<1)	0	0	3 (2)
Appetite disorder	1 (<1)	1 (<1)	0	0	2 (1)
Back pain	1 (<1)	1 (<1)	0	0	2 (1)
Clostridium difficile colitis	0	1 (<1)	1 (<1)	0	2 (1)
Dysgeusia	2 (1)	0	0	0	2 (1)
Dysphonia	2 (1)	0	0	0	2 (1)
Enterocolitis	0	1 (<1)	1 (<1)	0	2 (1)
Immune-mediated hepatitis	0	0	2 (1)	0	2 (1)
Immune-mediated thyroiditis	1 (<1)	1 (<1)	0	0	2 (1)
Nasal congestion	1 (<1)	1 (<1)	0	0	2 (1)
Neck pain	1 (<1)	1 (<1)	0	0	2 (1)
Night sweats	2 (1)	0	0	0	2 (1)
Pain in extremity	1 (<1)	1 (<1)	0	0	2 (1)
Platelet count decreased	1 (<1)	1 (<1)	0	0	2 (1)
Productive cough	1 (<1)	1 (<1)	0	0	2 (1)
Rash macular	1 (<1)	1 (<1)	0	0	2 (1)
Thyroiditis	0	2 (1)	0	0	2 (1)
Abdominal distension	0	1 (<1)	0	0	1 (<1)
Acute respiratory distress syndrome	0	1 (<1)	0	0	1 (<1)
Arthritis	1 (<1)	0	0	0	1 (<1)
Asthenia	1 (<1)	0	0	0	1 (<1)
Autoimmune colitis	0	1 (<1)	0	0	1 (<1)

Bell's Palsy	0	0	1 (<1)	0	1 (<1)
Blood thyroid stimulating hormone decreased	1 (<1)	0	0	0	1 (<1)
C-reactive protein increased	1 (<1)	0	0	0	1 (<1)
Constipation	1 (<1)	0	0	0	1 (<1)
Contusion	1 (<1)	0	0	0	1 (<1)
Dermatitis bullous	0	1 (<1)	0	0	1 (<1)
Dermatitis psoriasiform	0	1 (<1)	0	0	1 (<1)
Dysuria	1 (<1)	0	0	0	1 (<1)
Face edema	0	1 (<1)	0	0	1 (<1)
Facial nerve disorder	0	1 (<1)	0	0	1 (<1)
Flatulence	1 (<1)	0	0	0	1 (<1)
Flushing	1 (<1)	0	0	0	1 (<1)
Gastrointestinal pain	1 (<1)	0	0	0	1 (<1)
Glossodynia	1 (<1)	0	0	0	1 (<1)
Granulomatous lymphadenitis	1 (<1)	0	0	0	1 (<1)
Hyperkalemia	0	1 (<1)	0	0	1 (<1)
Hyponatremia	1 (<1)	0	0	0	1 (<1)
Hypophysitis	0	1 (<1)	0	0	1 (<1)
Hypotension	0	1 (<1)	0	0	1 (<1)
Hypoxia	0	1 (<1)	0	0	1 (<1)
Immune-mediated hypohysitis	0	0	1 (<1)	0	1 (<1)
Immune-mediated hypothyroidism	0	1 (<1)	0	0	1 (<1)
Immune-mediated myocarditis	0	0	1 (<1)	0	1 (<1)
Influenza	0	1 (<1)	0	0	1 (<1)
Infusion-related hypersensitivity reaction	0	1 (<1)	0	0	1 (<1)
Injection site infection	0	1 (<1)	0	0	1 (<1)
Interleukin level increased	1 (<1)	0	0	0	1 (<1)
Joint stiffness	1 (<1)	0	0	0	1 (<1)
Libido decreased	1 (<1)	0	0	0	1 (<1)
Lip infection	1 (<1)	0	0	0	1 (<1)
Lymph node pain	1 (<1)	0	0	0	1 (<1)
Myocarditis	0	0	1 (<1)	0	1 (<1)
Non-cardiac chest pain	1 (<1)	0	0	0	1 (<1)
Peripheral edema	1 (<1)	0	0	0	1 (<1)
Oral candidiasis	0	1 (<1)	0	0	1 (<1)
Oral herpes	1 (<1)	0	0	0	1 (<1)
Platelet count increased	1 (<1)	0	0	0	1 (<1)
Polyarthritis	0	0	1 (<1)	0	1 (<1)
Proteinuria	1 (<1)	0	0	0	1 (<1)
Pulmonary hypertension	0	1 (<1)	0	0	1 (<1)
Rash papular	1 (<1)	0	0	0	1 (<1)
Rash pustular	1 (<1)	0	0	0	1 (<1)
Red blood cell sedimentation rate increased	1 (<1)	0	0	0	1 (<1)
Right ventricular dysfunction	0	0	1 (<1)	0	1 (<1)
Sinus tachycardia	1 (<1)	0	0	0	1 (<1)
Skin exfoliation	1 (<1)	0	0	0	1 (<1)
Skin lesion	1 (<1)	0	0	0	1 (<1)
Triiodothyronine increased	1 (<1)	0	0	0	1 (<1)
Troponin increased	1 (<1)	0	0	0	1 (<1)
Troponin T increased	0	0	1 (<1)	0	1 (<1)
Vision blurred	0	1 (<1)	0	0	1 (<1)
White blood cell count increased	1 (<1)	0	0	0	1 (<1)

[a]Treatment-emergent adverse events (TEAEs) were coded using the Medical Dictionary Regulatory Activities, Version 22.1, classified by system organ class and preferred term. [b]Treatment-emergent adverse events related to botensilimab (BOT) or balstilimab (BAL) were coded using the Medical Dictionary Regulatory Activities, Version 22.1, classified by system organ class and preferred term. Treatment-related adverse events (TRAEs; TEAEs related to BOT or BAL) were defined as adverse events with onset dates or the worsening of an event

during the extended on-treatment period, which was defined as time from the first dose of study treatment to last dose of study treatment +90 days, or the earliest date of new anticancer therapy –1 day, whichever occurred first.

Supplementary Table 6 I Summary of immune-mediated adverse events and listing of all treatment-related immune-mediated adverse events in all treated patients with microsatellite stable metastatic colorectal cancer (*N*=148)

	**All treated** **_N_=148**
**Any imAE [a], *n* (%)**	78 (53)
Grade 1	7 (5)
Grade 2	37 (25)
Grade 3	32 (22)
Grade 4	2 (1)
Grade 5	0
imAE leading to treatment interruption	37 (25)
Grade 1	2 (1)
Grade 2	21 (14)
Grade 3	13 (9)
Grade 4	1 (<1)
Grade 5	0
imAE leading to permanent treatment discontinuation	36 (24)
Grade 1	2 (1)
Grade 2	16 (11)
Grade 3	18 (12)
Grade 4	0
Grade 5	0
imAE treated with systemic corticosteroids	71 (48)
Grade 1	6 (4)
Grade 2	34 (23)
Grade 3	29 (20)
Grade 4	2 (1)
Grade 5	0
imAE treated with immunosuppressants	44 (30)
Grade 1	5 (3)
Grade 2	23 (16)
Grade 3	15 (10)
Grade 4	1 (<1)
Grade 5	0

Immune-mediated TRAE Listing by Grade<br>*N*=148					
	**Grade 1**	**Grade 2**	**Grade 3**	**Grade 4**	**All-grade**
**Any immune-mediated TRAE[a], *n* (%)**	4 (3)	40 (27)	26 (18)	2 (1)	72 (49)
Diarrhea/colitis[b]	2 (1)	31 (21)	15 (10)	1 (<1)	49 (33)
Skin adverse reaction[c]	16 (11)	10 (7)	0	0	26 (18)
Hepatitis[d]	9 (6)	5 (3)	4 (3)	0	18 (12)
Other immune-mediated reactions[e]	8 (5)	5 (3)	1 (<1)	0	14 (9)
Pneumonitis[f]	1 (<1)	7 (5)	1 (<1)	0	9 (6)
Thyroiditis[g]	5 (3)	3 (2)	0	0	8 (5)
Hypothyroidism[h]	0	7 (5)	0	0	7 (5)
Myositis/rhabdomyolysis[i]	2 (1)	1 (<1)	1 (<1)	1 (<1)	5 (3)
Nephritis and renal dysfunction[j]	2 (1)	1 (<1)	2 (1)	0	5 (3)
Adrenal insufficiency[k]	0	2 (1)	1 (<1)	0	3 (2)
Hyperthyroidism[l]	3 (2)	0	0	0	3 (2)
Pancreatitis[m]	0	2 (1)	1 (<1)	0	3 (2)
Nervous system disorder[n]	1 (<1)	1 (<1)	0	0	2 (1)
Ocular disorder[o]	2 (1)	0	0	0	2 (1)
Myocarditis/pericarditis[p]	0	0	1 (<1)	0	1 (<1)
Pituitary dysfunction[q]	0	0	1 (<1)	0	1 (<1)

[a]Treatment-emergent adverse events (TEAEs) related to botensilimab (BOT) or balstilimab (BAL) were coded using the Medical Dictionary for Regulatory Activities, version 22.1, classified by system organ class and preferred term. Treatment-related adverse events (TRAEs; TEAEs related to BOT or BAL) were defined as adverse events with onset dates or the worsening of an event during the extended on-treatment period, which was defined as time from the first dose of study treatment to last dose of study treatment +90 days, or the earliest date of new anticancer therapy −1 day, whichever occurred first. Immune-mediated adverse events (imAEs) were defined as TEAEs that were treated with steroids or other immunosuppressants. Immune-mediated TRAEs were defined as TEAEs related to BOT or BAL that were treated with steroids or other immunosuppressants. [b]Immune-mediated diarrhea/colitis is comprised of the following preferred terms: colitis, diarrhea, and enteritis. [c]Immune-mediated skin adverse reaction is comprised of the following preferred terms: pruritis, rash, rash maculopapular, and eczema. [d]Immune-mediated hepatitis is comprised of the following preferred terms: alanine aminotransferase increased, aspartate aminotransferase increased, blood bilirubin increased, hepatitis, and immune-mediated hepatitis. [e]Other immune-mediated reactions is comprised of the following preferred terms: nausea, vomiting, stomatitis, gastritis, generalized edema, and polyarthritis. [f]Immune-mediated pneumonitis is comprised of the following preferred terms: pneumonitis and dyspnea. [g]Immune-mediated thyroiditis is comprised of the following preferred terms: blood thyroid stimulating hormone increased, thyroiditis, and blood thyroid stimulating hormone decreased. [h]Immune-mediated hypothyroidism is comprised of the following preferred term: hypothyroidism. [i]Immune-mediated myositis/rhabdomyolysis is comprised of the following preferred terms: blood creatine phosphokinase increased and myositis. [j]Immune-mediated nephritis and renal dysfunction is comprised of the following preferred terms: blood creatinine increased and acute kidney injury. [k]Immune-mediated adrenal insufficency is comprised of the following preferred term: adrenal insufficiency. [l]Immune-mediated hyperthyroidism is comprised of the following preferred term: hyperthroidism. [m]Immune-mediated pancreatitis is comprised of the following preferred terms: amylase increased and lipase increased. [n]Immune-mediated nervous system disorder is comprised of the following preferred terms: facial nerve disorder, glossodynia, and neuropathy peripheral. [o]Immune-mediated ocular disorder is comprised of the following preferred term: conjunctivitis. [p]Immune-mediated myocarditis/pericarditis is comprised of the following preferred term: myocarditis. [q]Immune-mediated pituitary dysfunction is comprised of the following preferred term: hypophysitis.

Supplementary Table 7 | Individual patient genomics and biomarker data

(***please see separate attachment due to size***)

Supplementary Table 8 | Gene signatures used in analysis of RNASeq data

**Signature**	**Signature gene list**
Angiogenesis (Bagaev et al.)[32]	*VEGFA, VEGFB, VEGFC, PDGFC, CXCL8, CXCR2, FLT1, PGF, CXCL5, KDR, ANGPT1, ANGPT2, TEK, VWF, CDH5*
Antitumor cytokines (Bagaev et al.)[32]	*TNF, IFNB1, IFNA2, CCL3, TNFSF10, IL21*
B cells (Bagaev et al.)	*CD19, MS4A1, TNFRSF13C, CR2, TNFRSF17, TNFRSF13B, CD22, CD79A, CD79B, BLK, FCRL5, PAX5, STAP1*
Cancer-associated fibroblasts (Bagaev et al.)	*COL1A1, COL1A2, COL5A1, ACTA2, FGF2, FAP, LRP1, CD248, COL6A1, COL6A2, COL6A3, CXCL12, FBLN1, LUM, MFAP5, MMP3, MMP2, PDGFRB, PDGFRA*
Checkpoint molecules (Bagaev et al.)	*PDCD1, CD274, CTLA4, LAG3, PDCD1LG2, BTLA, HAVCR2, TIGIT, VSIR, C10orf54*
Co-activation molecules (Bagaev et al.)	*CD28, CD40, TNFRSF4, ICOS, TNFRSF9, CD27, CD80, CD86, CD40LG, CD83, TNFSF4, ICOSLG, TNFSF9, CD70*
EMT signature (Bagaev et al.)	*SNAI1, SNAI2, TWIST1, TWIST2, ZEB1, ZEB2, CDH2*
Effector cell traffic (Bagaev et al.)	*CXCL9, CXCL10, CXCL11, CX3CL1, CCL3, CCL4, CX3CR1, CCL5, CXCR3*
Effector cells (Bagaev et al.)	*IFNG, GZMA, GZMB, PRF1, GZMK, ZAP70, GNLY, FASLG, TBX21, EOMES, CD8A, CD8B*
Endothelium (Bagaev et al.)	*NOS3, KDR, FLT1, VCAM1, VWF, CDH5, MMRN1, ENG, CLEC14A, MMRN2*
Granulocyte traffic (Bagaev et al.)	*CXCL8, CXCL1, CXCL2, CXCL5, CCL11, KITLG, CXCR1, CXCR2, CCR3*
IFNγ (Ayers et al.)[31]	*IFNG, STAT1, CXCL9, CXCL10, IDO1, HLA-DRA*
M1 signature (Bagaev et al.)	*NOS2, TNF, IL1B, SOCS3, CMKLR1, IRF5, IL12A, IL12B, IL23A*
MHCI (Bagaev et al.)	*HLA-A, HLA-B, HLA-C, B2M, TAP1, TAP2, TAPBP*
MHCII (Bagaev et al.)	*HLA-DRA, HLA-DRB1, HLA-DMA, HLA-DPA1, HLA-DPB1, HLA-DMB, HLA-DQB1, HLA-DQA1, CIITA*
Macrophage and DC traffic (Bagaev et al.)	*CCL2, CCL7, CCL8, XCL1, CCR2, XCR1, CSF1R, CSF1*
Matrix (Bagaev et al.)	*FN1, COL1A1, COL1A2, COL4A1, COL3A1, VTN, LGALS7, LGALS9, LAMA3, LAMB3, LAMC2, TNC, ELN, COL5A1, COL11A1*
Matrix remodeling (Bagaev et al.)	*CA9, MMP9, MMP2, MMP1, MMP3, MMP12, MMP7, MMP11, PLOD2, ADAMTS4, ADAMTS5, LOX*
Myeloid cells (Bagaev et al.)	*IDO1, ARG1, IL10, CYBB, PTGS2, IL4I1, IL6*
Myeloid cells traffic (Bagaev et al.)	*CSF2, CSF3, CXCL12, CCL26, IL6, CXCL8, CXCL5, CSF1R, CSF2RA, CSF3R, CXCR4, IL6R, CXCR2, CCL15, CSF1*
NK cells (Bagaev et al.)	*NKG7, CD160, CD244, NCR1, KLRC2, KLRK1, CD226, GZMH, GNLY, IFNG, KIR2DL4, EOMES, GZMB, FGFBP2, KLRF1, SH2D1B, NCR3*
Neutrophil signature (Bagaev et al.)	*MPO, ELANE, PRTN3, CTSG, CXCR1, CXCR2, FCGR3B, CD177, FFAR2, PGLYRP1*
Protumor cytokines (Bagaev et al.)	*IL10, TGFB1, TGFB2, TGFB3, IL22, MIF, IL6*
T cells (Bagaev et al.)	*TBX21, ITK, CD3D, CD3E, CD3G, TRAC, TRBC1, TRBC2, CD28, CD5, TRAT1*

Th1 signature (Bagaev et al.)	*IFNG, IL2, CD40LG, IL21, TBX21, STAT4, IL12RB2*
Th2 signature (Bagaev et al.)	*IL4, IL5, IL13, IL10, GATA3, CCR4*
Treg (Bagaev et al.)	*FOXP3, CTLA4, IL10, TNFRSF18, CCR8, IKZF4, IKZF2*
Treg and Th2 traffic (Bagaev et al.)	*CCL17, CCL22, CCL1, CCL28, CCR4, CCR8, CCR10*
Tumor proliferation rate (Bagaev et al.)	*MKI67, ESCO2, CETN3, CDK2, CCND1, CCNE1, AURKA, AURKB, E2F1, MYBL2, BUB1, PLK1, CCNB1, MCM2, MCM6*
Tumor-associated macrophages (Bagaev et al.)	*IL10, MRC1, MSR1, CD163, CSF1R, IL4I1, SIGLEC1, CD68*

EMT, epithelial-to-mesenchymal transition; IL, interleukin; MHC, major histocompatibility; NK, natural killer; Th, T helper cell; T_{reg}, regulatory T cells.

Supplementary Table 9 | Summary of major changes for each protocol amendment

Protocol	Date	List of major changes

**amendment**		
**Amendment 1**	January 10, 2019	• Dose rationale for BOT modified<br>• Additional information on DLTs included<br>• Study drug storage and preparation information added
**Amendment 2**	May 14, 2019	• Modified inclusion and exclusion criteria to provide clarification<br>• Modified dosing so that BOT is administered as a 60 (-10/+20) min IV infusion for all doses<br>• Changed tumor assessments to be performed Q6W (± 3 days) until treatment discontinuation in order to collect more comprehensive and consistent tumor response data<br>• Modified the Schedule of Assessments for PK data to reduce the number of blood samples<br>• Restructured the follow-up period to include a 90-day safety follow-up visit and a survival follow-up and remove unnecessary clinic visits for the convenience of patients<br>• Revised the events that are considered DLTs, criteria for treatment delay, and criteria for discontinuation of study treatment for clarity.<br>• Modified treatment beyond disease progression
**Amendment 3**	October 17, 2019	• Added two treatment arms: BOT Q6W monotherapy and a Q6W combination therapy arm with BOT and BAL<br>• A fresh tumor biopsy will now be required at baseline and on treatment
**Amendment 4**	July 6, 2020	• Added option for those experiencing PD in the monotherapy treatment arms to transition to combination therapy<br>• Flexibility added to explore additional dose levels and indications of interest<br>• Removed the third cohort from the accelerated dose escalation and added the third cohort to the 3+3 design<br>• Dosing for cohort 3 in the Q6W Monotherapy arm changed<br>• Discretionary imaging at 90-day survival follow-up and duration/criteria added<br>• DLT observation period for the first patient updated to 11 days across all cohorts
**Amendment 5**	October 1, 2021	• Safety Monitoring Committee decided to add an additional cohort, cohort 6, to the Q6W combination therapy treatment arm. This cohort is to receive an additional cohort of up to 20 patients (Cohort 6) investigating

		the following doses/schedule: BOT 150 mg administered on day 1 of each 6-week cycle in combination with BAL 450 mg administered on day 1 and day 22 of each 6-week cycle<br>• Table 9 was added to elaborate on the dosing cohorts in the 6-week combination therapy treatment arm<br>• AGEN2034 and AGEN1884 were updated to balstilimab and zalifrelimab, respectively<br>• The overall number of patients and investigative sites was increased to 125 and 15, respectively
**Amendment 6**	March 28, 2022	• Provided for additional subjects to be enrolled<br>• Deleted of Adverse Events of Special Interest (AESI) Section<br>• Updated the management of immune-related adverse events (irAEs)
**Amendment 7**	March 24, 2023	• Updated patient numbers<br>• Refined eligibility criteria for selected tumor types<br>• Updated toxicity management language for immune-related diarrhea/colitis<br>• As no further enrollment in the 6-week monotherapy cohort is anticipated, all details pertaining to 6-week monotherapy (eg, Schedule of Assessments) were removed (except for statistical components)<br>• Dose escalation has completed so study design and operational elements pertaining to dose-escalation have been removed to increase readability for new and future study sites
**Amendment 7.1**	July 7, 2023	• Country-specific amendment applicable to sites in the United Kingdom (UK<br>• Addressed a request made by the UK regulatory authority (Medicines and Healthcare Products Regulatory Agency) to clarify post-study availability of the study treatments
**Amendment 8**	October 5, 2023	• Increased the number of patients in the NSCLC refractory to PD-(L)1 inhibitor treatment cohort (60 additional patients added), for a total of 550 planned patients in the trial

Supplementary Figure 1 I Characteristics of patients with microsatellite stable metastatic colorectal cancer with stable disease or progressive disease by liver involvement (*n*=74). No active liver metastases (NLM) were defined as patients with no history of liver metastases (LM) or patients whose LM were resected or ablated without

recurrence. (a) Patients with stable disease (SD) and no history of LM (*n*=28); (b) patients with SD and treated LM (*n*=9); (c) patients with SD with active LM (*n*=6); (d) patients with progressive disease (PD) and no history of LM (*n*=12); (e) patients with PD and treated LM (*n*=4); (f) patients with PD and active LM (*n*=15). Arrows indicate patients who were ongoing on treatment at the time of data cutoff. [a]One patient had a response by Response Evaluation Criteria in Solid Tumors version 1.1 (RECIST 1.1) through week 66, at which time the patient developed a peri-splenic nodule that was assessed as a new lesion despite being present since week 18 when the initial response occurred (identified retrospectively); despite continued response in other lesions, the response was considered PD by RECIST 1.1. [b]Another patient had a tumor reduction including a reduction in lung metastases; the patient then had the primary tumor resected, which showed a complete pathologic response and subsequently was non-evaluable by imaging criteria. [c]Another patient had a PR in target lesions but experienced recurrence of treated LM at week 18. BOT, botensilimab.

a

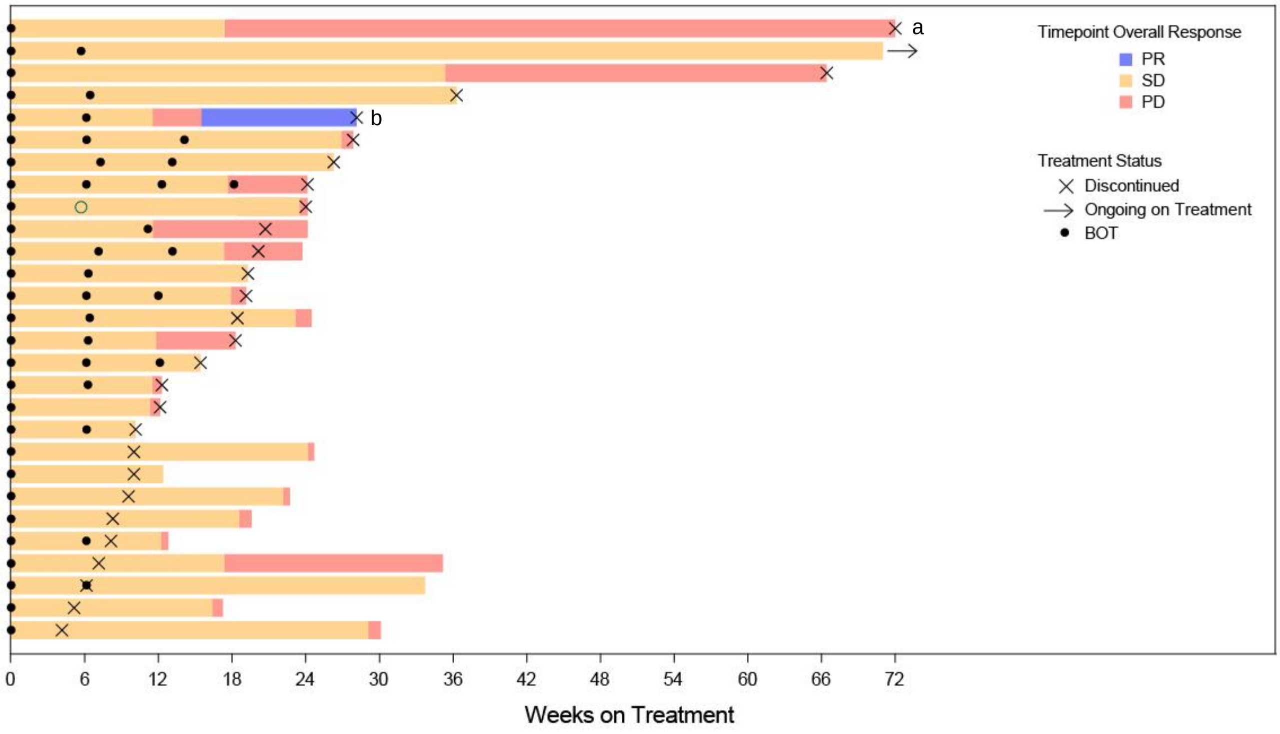

b

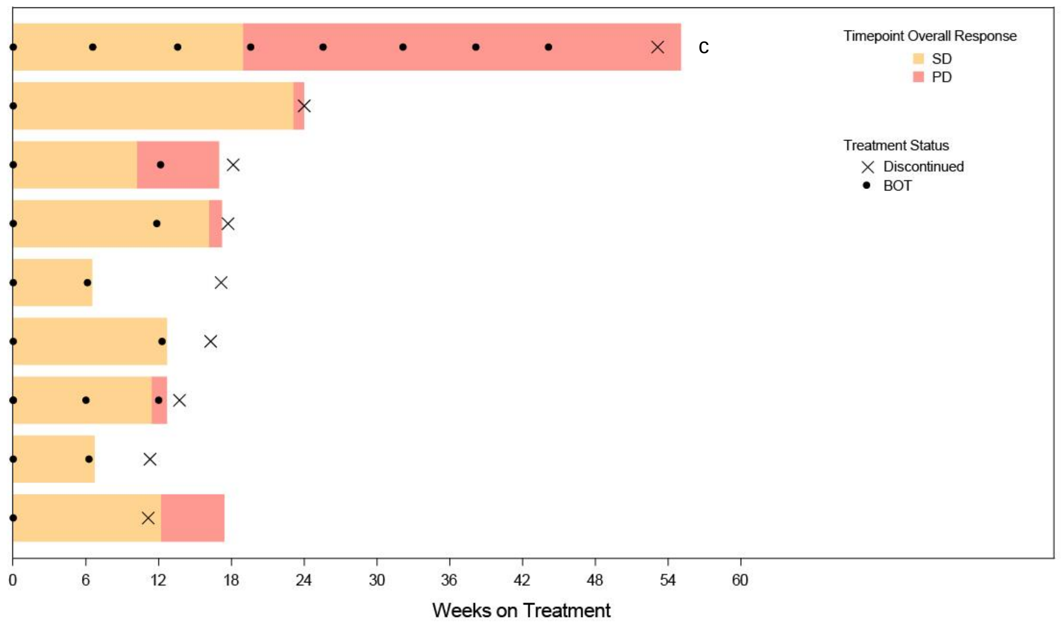
c
Timepoint Overall Response
SD
PD
Treatment Status
Discontinued
BOT
0
6
12
18
24
30
36
42
48
54
60
Weeks on Treatment

c

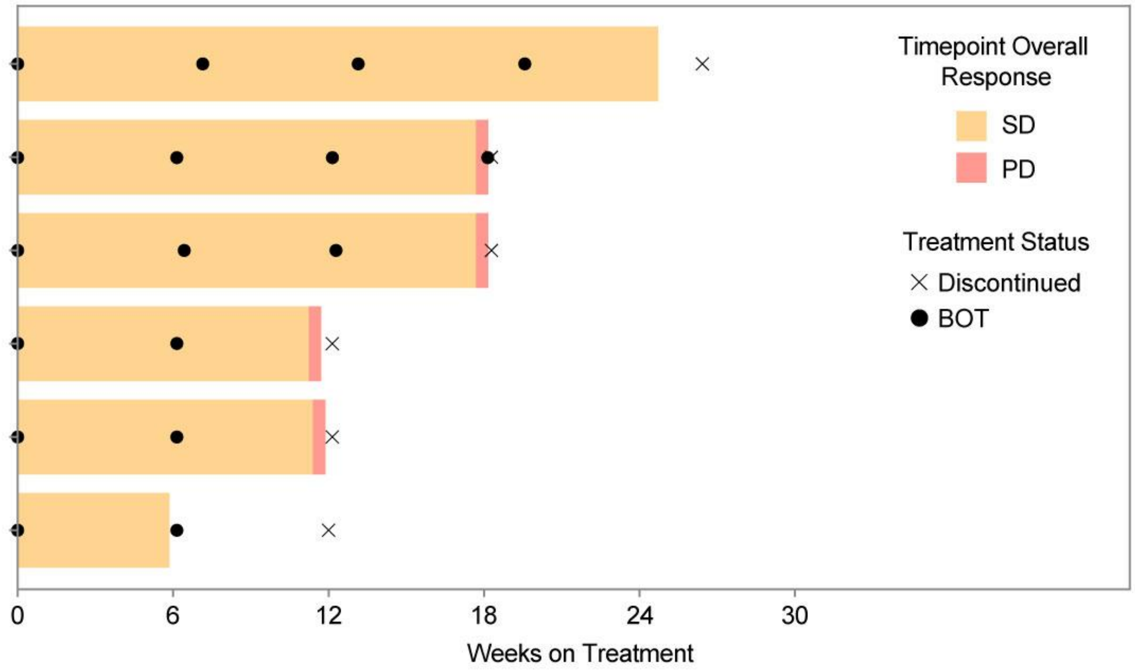
Timepoint Overall Response
SD
PD
Treatment Status
Discontinued
BOT
0
6
12
18
24
30
Weeks on Treatment

d

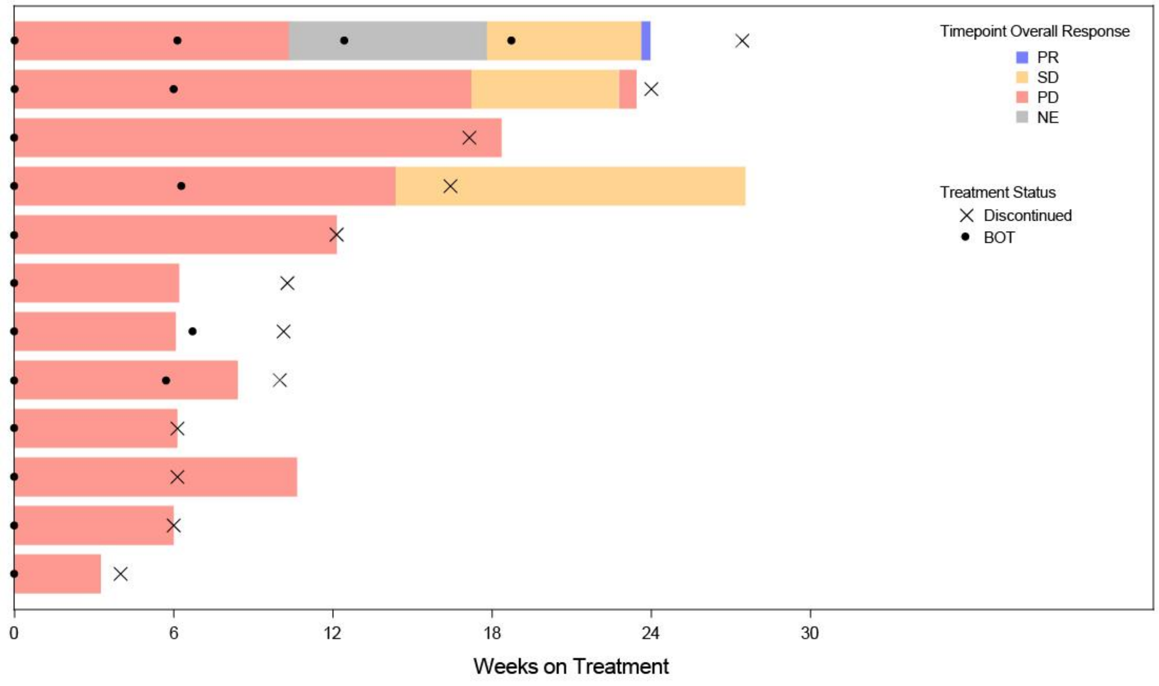
Timepoint Overall Response
PR
SD
PD
NE
Treatment Status
Discontinued
BOT
0
6
12
18
24
30
Weeks on Treatment

e

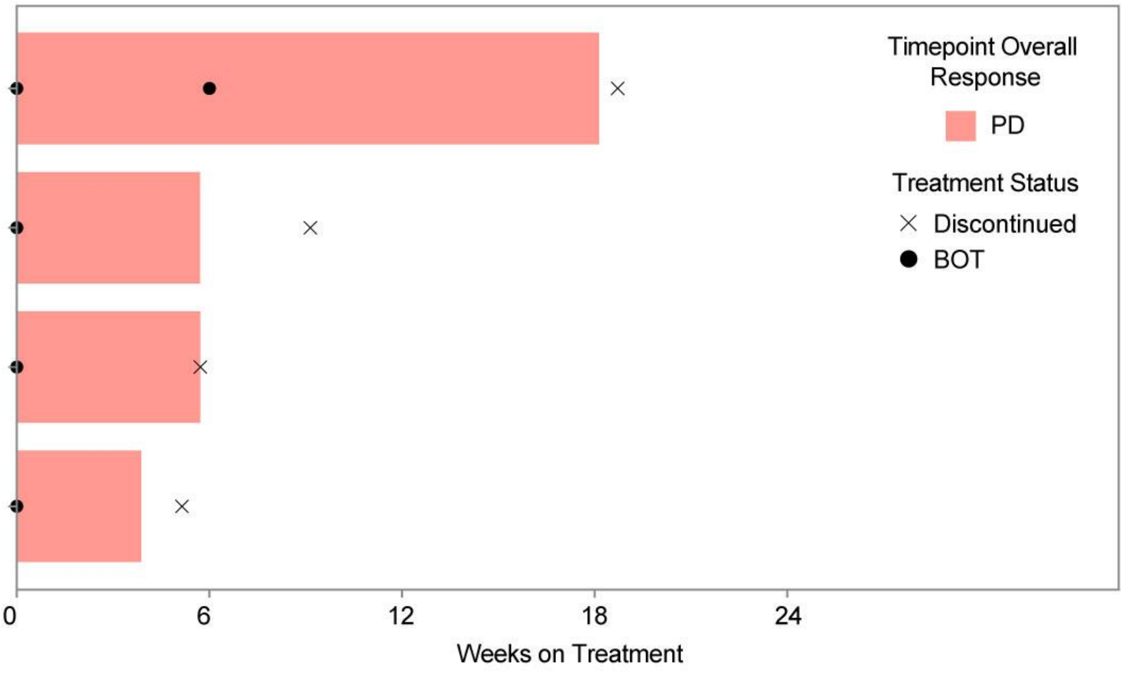
Timepoint Overall Response
PD
Treatment Status
Discontinued
BOT
0
6
12
18
24
Weeks on Treatment

f

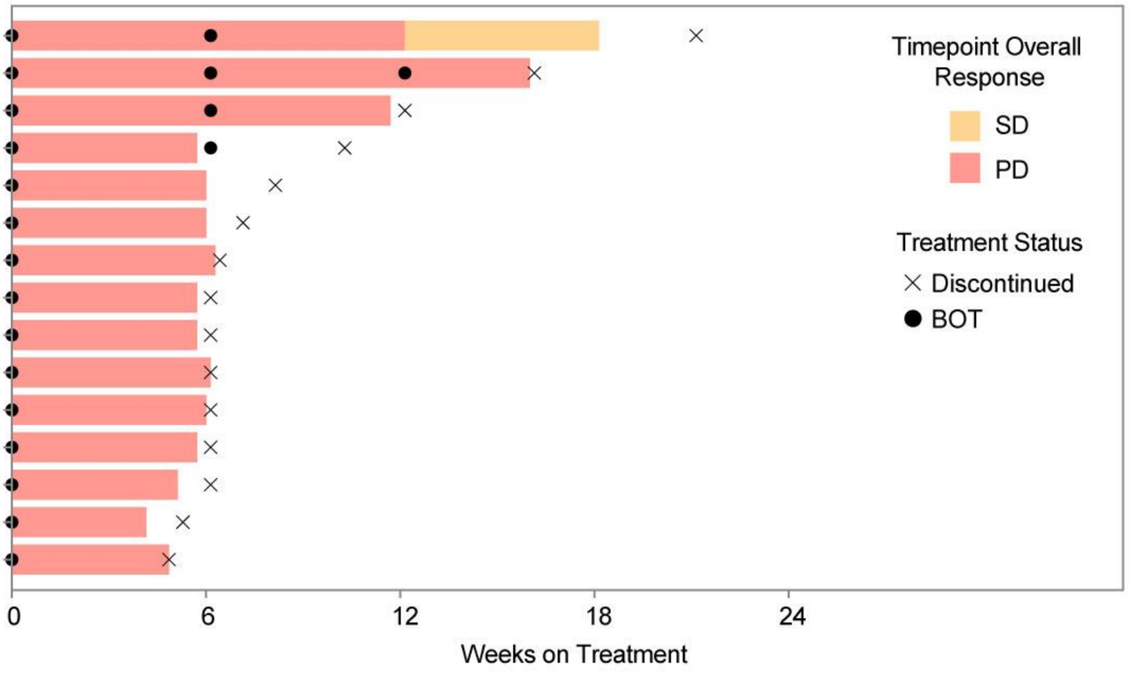
Timepoint Overall Response
SD
PD
Treatment Status
Discontinued
BOT
0
6
12
18
24
Weeks on Treatment
