# Supplementary material for: Botensilimab plus balstilimab in relapsed/refractory microsatellite stable metastatic colorectal cancer: a phase 1 trial
Source: Nat Med. 2024 Jun 13;30(9):2558–67. doi: 10.1038/s41591-024-03083-7 (PMC11405281; doi:10.1038/s41591-024-03083-7)
Supplement: Supplementary file 2 — Reporting Summary [file 41591_2024_3083_MOESM2_ESM.pdf]

nature portfolio

Corresponding author(s): Anthony El-Khoueiry and Justin Stebbing
Last updated by author(s): May 18, 2024

Reporting Summary

Nature Portfolio wishes to improve the reproducibility of the work that we publish. This form provides structure for consistency and transparency in reporting. For further information on Nature Portfolio policies, see our Editorial Policies and the Editorial Policy Checklist.

Statistics

For all statistical analyses, confirm that the following items are present in the figure legend, table legend, main text, or Methods section.

n/a	Confirmed	
☐	☒	The exact sample size (*n*) for each experimental group/condition, given as a discrete number and unit of measurement
☒	☐	A statement on whether measurements were taken from distinct samples or whether the same sample was measured repeatedly
☐	☒	The statistical test(s) used AND whether they are one- or two-sided<br>*Only common tests should be described solely by name; describe more complex techniques in the Methods section.*
☒	☐	A description of all covariates tested
☒	☐	A description of any assumptions or corrections, such as tests of normality and adjustment for multiple comparisons
☐	☒	A full description of the statistical parameters including central tendency (e.g. means) or other basic estimates (e.g. regression coefficient) AND variation (e.g. standard deviation) or associated estimates of uncertainty (e.g. confidence intervals)
☐	☒	For null hypothesis testing, the test statistic (e.g. *F*, *t*, *r*) with confidence intervals, effect sizes, degrees of freedom and *P* value noted<br>*Give P values as exact values whenever suitable.*
☒	☐	For Bayesian analysis, information on the choice of priors and Markov chain Monte Carlo settings
☒	☐	For hierarchical and complex designs, identification of the appropriate level for tests and full reporting of outcomes
☒	☐	Estimates of effect sizes (e.g. Cohen's *d*, Pearson's *r*), indicating how they were calculated

Our web collection on statistics for biologists contains articles on many of the points above.

Software and code

Policy information about availability of computer code

Data collection	NovaSeq 6000 and NovaSeq X Plus sequencing platforms
Data analysis	Somatic variants were called from the exome reads and reference human genome using hs37d5 and BWA (https://hpc.nih.gov/apps/bwa.html#:~:text=BWA%20excels%20in%20its%20speed,length%20and%20the%20maximum%20mismatches.) and STAR alignment tools (https://support.illumina.com/help/BS_App_RNASeq_Alignment_OLH_1000000006112/Content/Source/Informatics/STAR_RNAseq.htm), and variants were called using MuTect (https://gatk.broadinstitute.org/hc/en-us/articles/360037593851-Mutect2), Verdict, and Fusion-Catcher (https://github.com/ndaniel/fusioncatcher) as part of Personalis ImmunoID NeXT analysis pipeline (https://www.personalis.com/for-biopharma/immunoid-next/).<br><br>For RNA-seq: Bulk RNA-Seq expression data was derived from pretreatment samples and the log2 gene transcripts per million (TPM) counts were transformed to Z-score values. Gene signatures were then calculated by averaging gene expression values sample-wise and the results were compared across outcomes (CR or PR, SD≥12 weeks, or PD). R version 4.1.2 (2021-11-01) and additional R libraries (dplyr_1.1.4, ggplot2_3.5.0, and FSA_0.9.4) were used for gene expression analysis by outcome.

For manuscripts utilizing custom algorithms or software that are central to the research but not yet described in published literature, software must be made available to editors and reviewers. We strongly encourage code deposition in a community repository (e.g. GitHub). See the Nature Portfolio guidelines for submitting code & software for further information.

Data

Policy information about availability of data

All manuscripts must include a data availability statement. This statement should provide the following information, where applicable:

- Accession codes, unique identifiers, or web links for publicly available datasets
- A description of any restrictions on data availability
- For clinical datasets or third party data, please ensure that the statement adheres to our policy

Deidentified individual participant clinical data, WES and bulk RNA-Seq data that underlie the results reported in this article are available for transfer upon request for academic use and within the limitations of the provided informed consent. Interested investigators can obtain and certify the data transfer agreement and submit requests to the corresponding author (A.B. El-K. [elkhouei@med.usc.edu]). Investigators who consent to the terms of the data transfer agreement, including, but not limited to, the use of these data only for research purposes, and to protect the confidentiality of the data and limit the possibility of identification of patients in any way whatsoever for the duration of the agreement, will be granted access. Data will be available for request for a period of 2 years after the completion of the C-800-01 study. Requests will be evaluated on a case-by-case basis for a period of at most 2 weeks before a receipt of a response.

Research involving human participants, their data, or biological material

Policy information about studies with human participants or human data. See also policy information about sex, gender (identity/presentation), and sexual orientation and race, ethnicity and racism.

Reporting on sex and gender

The C-800-01 study recruited both males and females. The study did not collect gender-related data beyond the male-female dichotomy, which was self-reported. Gender-based outcomes were not relevant to the study objectives and thus data have not been disaggregated by gender except in a subsequent subanalysis (which will be the subject of a future manuscript). Anticancer activity was observed in both genders.

In the 83 patients from the dose escalation portion of the study, median age in all monotherapy cohorts was 66 years (range 31-81), and 62 years in all combination cohorts (range 25-82). In all monotherapy cohorts, there were 20 females and 28 males, and in all combination cohorts, there were 24 females and 11 males.

In the 148 all treated patients with MSS mCRC, median age was 56 years (range 25-82) and 76 patients were female and 72 were male. Gender distribution was thus balanced.

Patients were not compensated except for travel (granted upon request).

Reporting on race, ethnicity, or other socially relevant groupings

The C-800-01 study recruited patients of all races and ethnicities. Race and ethnicity-based outcomes and specific data were not relevant to the study objectives. Median patient age varied among phase 1 dose escalation patients, and the 148 all treated patients with MSS mCRC. Most patients had an Eastern Cooperative Oncology Group (ECOG) performance status of 1. The population was heavily pretreated with a median of 3 (range 0-10) prior therapies. A minority had received prior PD-(L)l and CTLA-4 inhibitors.

Population characteristics

Baseline demographics and disease history variables were collected and described to characterize the patient population.

Recruitment

Patients were recruited from the active sites in the United States (and later one in the United Kingdom), consisting of phase 1 centers, and later in the study, some GI-specific disease groups. During the initial portion of the study, all CRC patients were eligible; as responses were observed in patients with non-liver metastatic (NLM) disease, there was likely a bias by investigators towards enrolling these patients as they became aware of that data. Under a subsequent amendment, only patients with no history of liver metastases were enrolled. Finally under another later amendment, that restriction was lifted and patients enrolled to "cohort 2" were eligible even if they had treated liver metastases, but not active liver metastases. With regards to other sites of metastatic disease in NLM patients (which are historically associated with poor prognosis, e.g., peritoneum), these patients were heavily represented, and this may have been due to the unblinded nature of the study and investigators observing clinical benefit in these patients who have not historically benefited from I-O combinations. Finally, similar to most phase 1 trials for heavily pretreated patients, there was the potential to skew enrollment towards patients with more indolent disease who are able to enroll on phase 1 trials in a late line of therapy. While this could not be avoided, relevant baseline and disease characteristics such as time from diagnosis of metastatic disease to study enrollment were recorded.

Patients were recruited at fourteen different sites across the United States: The Angeles Clinic & Research Institute, a Cedars-Sinai Affiliate; Beth Israel Deaconess Medical Center; City of Hope Comprehensive Cancer Center; Columbia University Medical Center; Dana-Farber Cancer Institute; HonorHealth Research & Innovation Institute; MD Anderson Cancer Center; Memorial Sloan Kettering Cancer Center; Providence Portland Cancer Center; Saint John's Cancer Institute; University of Colorado; University of Miami Sylvester Comprehensive Cancer Center; University of Southern California Norris Comprehensive Cancer Center; and The University of Texas Health Science Center at San Antonio. All patients provided informed written consent.

Ethics oversight

Institutional review boards from fourteen sites across the United States approved this protocol.

Note that full information on the approval of the study protocol must also be provided in the manuscript.

Field-specific reporting

Please select the one below that is the best fit for your research. If you are not sure, read the appropriate sections before making your selection.

☒ Life sciences ☐ Behavioural & social sciences ☐ Ecological, evolutionary & environmental sciences

For a reference copy of the document with all sections, see nature.com/documents/nr-reporting-summary-flat.pdf

Life sciences study design

All studies must disclose on these points even when the disclosure is negative.

Sample size	In this dose escalation and dose expansion study, a cohort size of 40 in patients treated with the botensilimab and balstilimab combination was targeted in each of the selected tumor types to assess tumor response. The number of patients included in this study is sufficient for the evaluation of the botensilimab and balstilimab combination efficacy and safety as demonstrated in the manuscript.
Data exclusions	No data were excluded for patients in this report.
Replication	Data from this study is observational. Response is based on investigator assessment per RECIST v1.1 criteria.
Randomization	No analysis with covariate adjustment was performed since this was not a comparative trial.
Blinding	As this is an ongoing, open-label, phase 1a/1b trial, there was no blinding.

Reporting for specific materials, systems and methods

We require information from authors about some types of materials, experimental systems and methods used in many studies. Here, indicate whether each material, system or method listed is relevant to your study. If you are not sure if a list item applies to your research, read the appropriate section before selecting a response.

Materials & experimental systems

n/a	Involved in the study	
☒	☐	Antibodies
☒	☐	Eukaryotic cell lines
☒	☐	Palaeontology and archaeology
☒	☐	Animals and other organisms
☐	☒	Clinical data
☒	☐	Dual use research of concern
☒	☐	Plants

Methods

n/a	Involved in the study	
☒	☐	ChIP-seq
☒	☐	Flow cytometry
☒	☐	MRI-based neuroimaging

Clinical data

Policy information about clinical studies

All manuscripts should comply with the ICMJE guidelines for publication of clinical research and a completed CONSORT checklist must be included with all submissions.

Clinical trial registration	NCT03860272
Study protocol	Amendment 8 (current protocol version) will be available as part of the Supplementary Information.
Data collection	Data was collected via electronic database capture at fourteen sites across the United States. The date the first patient was enrolled was April 1, 2019. Data was collected from this time until November 29th, 2023 (at the time of the data cutoff).
Outcomes	Primary and secondary outcomes were defined in the original protocol, dated November 8, 2018.<br>Primary:<br>• Occurrence of DLT in subjects in dose escalation during the first 28 days of treatment<br>Secondary:<br>• Frequency, severity, and duration of TEAEs and laboratory abnormalities for all dose groups, according to NCI CTCAE version 5.0.<br>• PK profile of AGEN1181 and balstilimab.<br>• Immunogenicity of AGEN1181 and balstilimab.<br>• ORR, per RECIST 1.1 based on Investigator assessment.<br>• DOR, per RECIST 1.1 based on Investigator assessment.<br>• DCR (including complete and partial responders and SD), per RECIST 1.1 based on Investigator assessment.<br>• PFS per RECIST 1.1 based on Investigator assessment

Plants

Seed stocks	N/A
Novel plant genotypes	N/A
Authentication	N/A
